# A Gene-Phenotype Network Based on Genetic Variability for Drought Responses Reveals Key Physiological Processes in Controlled and Natural Environments

**DOI:** 10.1371/journal.pone.0045249

**Published:** 2012-10-08

**Authors:** David Rengel, Sandrine Arribat, Pierre Maury, Marie-Laure Martin-Magniette, Thibaut Hourlier, Marion Laporte, Didier Varès, Sébastien Carrère, Philippe Grieu, Sandrine Balzergue, Jérôme Gouzy, Patrick Vincourt, Nicolas B. Langlade

**Affiliations:** 1 INRA, Laboratoire des Interactions Plantes-Microorganismes (LIPM), UMR441, Castanet-Tolosan, France; 2 CNRS, Laboratoire des Interactions Plantes-Microorganismes (LIPM), UMR2594, Castanet-Tolosan, France; 3 INRA, Unité de Recherche en Génomique Végétale (URGV), UMR1165 – Université d'Evry Val d'Essonne - ERL CNRS 8196, CP 5708, Evry, France; 4 Université Toulouse, INPT ENSAT, UMR1248 AGIR, Castanet-Tolosan, France; 5 INRA, UMR1248 AGIR, Castanet-Tolosan, France; 6 INRA, AgroParisTech, UMR518, Paris, France; Ecole Normale Superieure, France

## Abstract

Identifying the connections between molecular and physiological processes underlying the diversity of drought stress responses in plants is key for basic and applied science. Drought stress response involves a large number of molecular pathways and subsequent physiological processes. Therefore, it constitutes an archetypical systems biology model. We first inferred a gene-phenotype network exploiting differences in drought responses of eight sunflower (*Helianthus annuus*) genotypes to two drought stress scenarios. Large transcriptomic data were obtained with the sunflower Affymetrix microarray, comprising 32423 probesets, and were associated to nine morpho-physiological traits (integrated transpired water, leaf transpiration rate, osmotic potential, relative water content, leaf mass per area, carbon isotope discrimination, plant height, number of leaves and collar diameter) using sPLS regression. Overall, we could associate the expression patterns of 1263 probesets to six phenotypic traits and identify if correlations were due to treatment, genotype and/or their interaction. We also identified genes whose expression is affected at moderate and/or intense drought stress together with genes whose expression variation could explain phenotypic and drought tolerance variability among our genetic material. We then used the network model to study phenotypic changes in less tractable agronomical conditions, i.e. sunflower hybrids subjected to different watering regimes in field trials. Mapping this new dataset in the gene-phenotype network allowed us to identify genes whose expression was robustly affected by water deprivation in both controlled and field conditions. The enrichment in genes correlated to relative water content and osmotic potential provides evidence of the importance of these traits in agronomical conditions.

## Introduction

Water scarcity, widely known as drought, is defined as the unbalance between the available water in the soil and the actual evaporative demand resulting from the climatic conditions [1]. This major environmental stress hinders plant growth and development as well as crop yield [2] . Moreover, water-limiting conditions will be increasingly common due to global warming and demographical pressure. As a result, water scarcity has been pointed out as the biggest agronomical problem worldwide, thus hampering food production in the future [3]. In this scenario, proper water management in agriculture is vital and, therefore, the use of crops that are capable of using water efficiently under a low input regime is a major farming objective.

Sunflower (*Helianthus annuus* L.) has been widely regarded as a plant able to grow under low water-input regimes. Besides, wild and domesticated *Helianthus annuus* ecotypes have successfully colonized most diverse climatic niches in North America, including harsh desert habitats, which indicates the richness of the gene pool of this species [4]–[6]. Nevertheless, sunflower genotypes are not homogeneously efficient in the use of water. In fact, this crop might on one hand waste water when this is available [7] and on the other hand maintain some productivity under when water is scarce. Furthermore, available soil water content and genotypic sensitivity to water status are interacting to influence plant development and productivity [8]. Thus, as the result of genotype * environment interaction under drought, the processes underpinning carbon assimilation, tissue expansion, biomass production and seed quality imply, among other regulatory mechanisms, the control of genes expression.

Several mechanisms help plants maintain their water status. First, at the plant organ level, the thickening of the cuticle, mainly by means of wax accumulation, helps reducing non-stomatal transpiration [9]. Then, decreasing the stomatal conductance remains the major short-term mechanism to limit water loss. The sooner the stomata close in response to water deficit, the longer the water potential in the leaves will be maintained. Stomatal closure may depend on the genotype [10], as well as on the developmental stage of the plant [11]. Another mechanism consists for plants to reduce their leaf surface and/or accelerate leaf senescence, reducing water loss and placing themselves in a more adapted phenotypic situation if the water stress goes on. At the cellular level, two factors determine leaf growth and expansion: cell wall extension and turgidity [12], [13]. Turgidity allows plants to carry on with their physiological functions under drought stress despite an eventual decrease in Relative Water Content (RWC) in the cells. Three mechanisms are involved in maintaining cell turgidity: osmotic adjustment by means of active osmolyte accumulation (essentially inorganic ions, soluble sugars, and carboxylic and amino acids), increasing cell wall elasticity and modifying water content repartition between the apoplast and the symplasm [14]. The ability of sunflower to manage osmotic adjustment in leaves depends on the genetic background [15]–[17], the characteristics of the water stress itself and the age of the leaf [18], [19].

Those diverse mechanisms demand tight genetic regulation. It has been described that thousands of Arabidopsis or rice genes are modulated in response to drought stress [20]. Not all of these genes are necessarily involved in drought tolerance: the modulation of expression of many of them under drought stress indirectly reflects the way the plant is coping with the stress. Moreover, genes that are modulated under water deprivation are not equally expressed or regulated during the whole duration of the stress [21]. Four distinct regulatory pathways controlling drought-responsive genes have been described, those pathways being either dependent on abscisic acid (ABA) or, on the contrary, ABA-independent [22], [23]. Thus, signal transduction mechanisms implemented under the perception of drought stress might be different according to the role that ABA might have in sensing the constraint factors [24]–[28].

The drought stress signal transduction pathways are complex and interconnected, involving not only ABA but also ethylene and jasmonate in Arabidopsis [29] and sunflower [30]. Furthermore, the downstream phenotypic responses at the molecular and physiological levels are numerous and driven by different signaling pathways. This complex system represents an archetypical model for network modeling approaches to embrace the global rules coordinating molecular processes and phenotypic responses during drought stress response.

Integrating and modeling protein biochemical and molecular functions, transcriptomic regulation during organism development and stress responses, and other genetic interactions can be achieved through graphs reviewed by Newman [31]. Resulting gene networks may be of various nature depending on the mathematical models they are based on, the nature of information used to generate them, and if they connect only genes or combine genes with phenotypic data and physiological processes. Expression data from microarray or second generation sequencing technologies allow the characterization of most if not all gene expression profiles according to genetic and/or environmental factors [32]. In this context, gene network inference has become widely used and allows the identification of central nodes or hubs that may serve as drivers in plant responses [33], [34] and “guilt-by-association” approaches to predict gene functions. However, solving direct versus indirect relationships in gene regulation is still challenging given the usually limited number of conditions tested, compared to the large number of gene assessed. Furthermore, the key functional genomics question of identifying relations between heterogeneous datasets such as gene expression and phenotypes has rarely been addressed in the past partly because of the lack of adapted biostatistical tools and the difficulty to run very computer-intensive statistical methods such as regularized Canonical Correlation Analysis [35] and partly because of the inherent difficulty of integrative biology approaches. Thanks to recent developments in sunflower genomics and performing mathematical tools, we present in this work, for the first time in plant biology, statistically integrated gene expression and phenotype data in an gene-phenotype network.

Besides the identification of gene functions and physiological traits in model plants under controlled conditions, another major objective of plant researchers is to transfer this knowledge to applied biological systems such as field crops to help breeding classical traits and develop new ones To date, only a few transcriptomic studies in field conditions have been published [36], [37], likely because of statistical issues due to the variability of environmental conditions. It is crucial to relate field condition studies and those performed under controlled conditions in greenhouses or growth chambers. Indeed, it remains central to know how far the key factors exhibited in controlled conditions are accounting for at least part of the plant responses in the field. Current statistical tools and genomics knowledge allowed us to pursue these approaches and combine results of drought stress responses both in controlled and natural environments.

Global transcriptomics and morpho-physiological phenotyping represent major sources of information in order to unravel gene networks accounting for drought responses in model and agronomic plants. However, such approaches have major caveats: genetic variability in drought sensitivity, and transferability to agronomic conditions. This is particularly the case for crops such as maize or sunflower, which are grown as hybrids in non-controlled field conditions. In this work, we exploit controlled-condition transcriptomics data to better understand the crop behavior under drought in natural environment.

By combining gene expression patterns and physiological descriptors in experiments revealing drought, genotypic and drought*genotypic effects, we produced gene-phenotype networks. This allowed us to disentangle the genetic and molecular mechanisms underpinning drought responses of sunflower in controlled conditions and to subject this model to agronomic reality.

## Results and Discussion

### Genotype-dependent water consumption

Eight genotypes were chosen for this study, paying attention to previous phenotyping data that provided evidence of genotype-dependent responses to different environmental cues, including water deprivation. SF193 (also known as XRQ) and SF326 (also known as PSC8) are parental lines of the “INEDI” RIL population developed by INRA [38], [39]. SF193 is a maintainer line whose pedigree includes the Progress cultivar, which improves the tolerance to Phomopsis and the resistance to Downy mildew, and the widely used HA89. Both SF193 and SF326 behaved differently in response to water deprivation in our preliminary studies. For instance, it was observed that SF193 closes its stomata at much lower water constraint in the soil than SF326 at the same developmental stage. INEDI, another genotype used in this work, corresponds to the F1 hybrid SF193*SF326. SF109, (also known as 2603) is an INRA-bred line that, despite its susceptibility to some diseases like Phomopsis, has been widely used as a female parental line in hybrids cultivated in Spain and other Southern European countries due to its good agronomic adaptation to dry conditions. Two other genotypes, SF028 and SF107, have previously been used as male parental lines in field test-crosses, and both show highly contrasted yields between irrigated and non-irrigated conditions, depending on the location. Finally, TEKNY and MELODY are widely cultivated sunflower hybrids.

**Figure 1:**
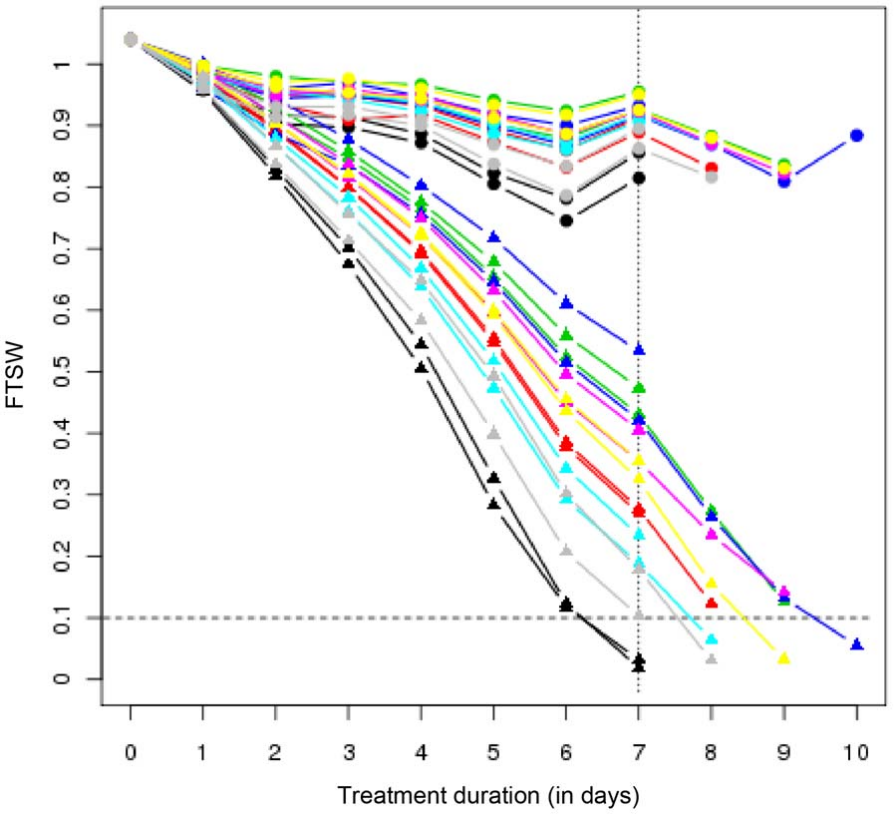
Evolution of Fraction of Transpirable Soil Water (FTSW) during water deprivation. Each line reflects the average values of three values for each genotype either under FDS or under FIS. Vertical dotted line indicates the date of the FDS tissue collection. Horizontal dotted line indicates the FTSW level at which FIS collection was carried out. Triangles correspond to treated plants whereas circles correspond to their untreated counterparts. Genotypes are color-coded as follows: Inedi (black), Tekny (gray), Melody (red), SF109 (turquoise), SF326 (yellow), SF193 (magenta), SF028 (green) and SF107 (blue).

Water irrigation of treated plants was stopped 25 days after sowing. From this moment on, the Fraction of Transpirable Soil Water (FTSW, chosen to reflect the soil water constraint) decreased differently according to the genotype in pots containing treated plants (see Fig. 1). The pace at which plants deplete their available water is directly related to their response to lack of irrigation. Our results show that all three hybrids (i.e INEDI, TEKNY and MELODY, in this order) are the genotypes that most hastily reduce their FTSW, along with line SF109. Then SF193 and SF326, the parental lines of INEDI, present similar water consumption, whereas FTSW in pots containing SF028 and SF107 plants decreases most slowly in our assay.

### Drought stress scenarios

The intrinsic genotypic differences in drought responses are difficult to unravel because they might be confounded with inherent differences in developmental stage. Hence, two distinct stress assessment scenarios were implemented. First, Fixed Duration Stress (FDS) was established to decipher the progressive sunflower response to water deprivation using genotypic differences in order to generate a range of timely comparable water constraints. Plants were thus harvested when 50% of the treated plants reached a FTSW below 0.35. This state happened to arrive seven days after stopping irrigation, when estimated FTSW values of the treated plants ranged from 0 to 0.57 (Table S1). Second, Fixed Intensity Stress (FIS) scenario was implemented with the purpose to unravel plant responses at a comparable, more severe drought constraint. Hence, tissue was collected from every treated plant and its corresponding control when the former reached an estimated FTSW value below 0.1, i.e. ranging from 0 to 0.09. In practice, this harvest was pursued over four days. Incidentally, both FDS and FIS harvests took place on the same day for INEDI plants.

In order to measure the extent of the water constraint under each scenario, the Integrated Transpired Water (ITW) variable was calculated by integrating the transpired water (i.e. 1-FTSW) over the treatment duration. Consequently, ITW under FDS reflects the transpired water at harvest day, whereas under FIS, it reflects treatment duration. (as shown in Fig. 1).

### Sunflower oligonucleotide array and HELIAGENE database

The HELIAGENE database (http://www.heliagene.org) hosts and curates the information concerning the assembly of 284 340 ESTs from seven different *Helianthus* species, mainly produced in frame of Compositae Genome Project (http://compgenomics.ucdavis.edu/compositae_index.php). From the resulting 87 237 clusters, 72 372 were predicted to encode a peptide using FrameDP [40], 24 799 of them being likely full length. This public tool allows multi-criteria searches based on, for instance, accession numbers, keywords, Gene Ontology (GO) terms, InterPro domains, Helianthus species similarities etc. It also permits BLAST queries and it offers several FASTA sequence-handling workflows in order to optimize different in silico studies.

The Affymetrix ® GeneChip ® WT array, which was built in the frame of a consortium associating L. Rieseberg at UBC (Vancouver, Canada), S. Knapp at UGA (Athens, Georgia, U.S.A), the companies BIOGEMMA and SYNGENTA Seeds, and INRA (France). It contains 2 389 915 probes whose sequences derive from the same 87 237 Helianthus EST clusters (7 species). For this study, even if we hybridized the entire chip and therefore all the probes, only probesets containing at least one Helianthus annuus EST were considered. By doing so, we aimed at (i) avoiding redundant transcripts in the analysis, which might have been clustered apart due to high polymorphism rate among the seven species used to generate the EST database and (ii) reducing hybridization noise due to high polymorphism between targets and probes. This led us to keep 32 423 probesets containing at least one Helianthus annuus EST. Overall, 897 642 probes were therefore considered, averaging 28 probes per probeset (Fig. S1).

This transcriptomic tool allowed us, under our experimental design, to perform (i) descriptive analysis of gene expression in different sunflower genotypes under distinct drought stress implementation scenarios; (ii) differential studies in order to determine factors altering gene transcription under such stress; and (iii) covariance analysis with the aim of establishing links between gene expression alterations and morpho-physiological variations.

### Global Comparison of FDS and FIS classifications

**Figure 2:**
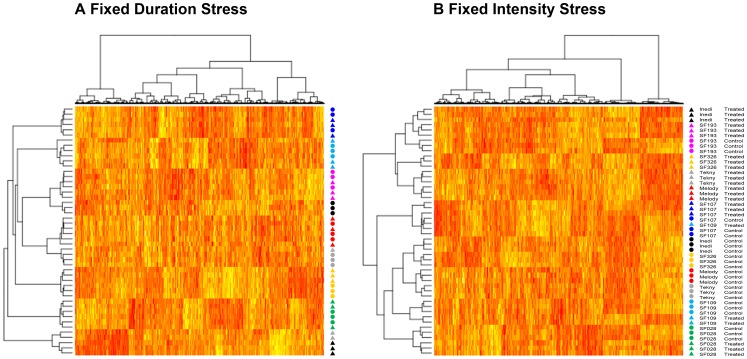
Dendograms and heatmaps of genes and individuals both under FDS (A) and FIS (B). Triangles correspond to treated plants whereas circles correspond to their untreated counterparts. Genotypes are color-coded as follows: Inedi (black), Tekny (gray), Melody (red), SF109 (turquoise), SF326 (yellow), SF193 (magenta), SF028 (green) and SF107 (blue).

Double hierarchical classifications of genes and individuals were performed independently for either stress implementation scenarios, taking into account only the genes going through statistically significant modulation under genotype, treatment or genotype*treatment (g*t) interaction (see below for an explanation on the Bonferroni-corrected ANOVA results). The resulting dendograms and subsequent heatmaps are shown in figure 2. It can be observed in figure 2 that individuals under FDS are mainly grouped by their genotype, with treated and control plants clustered together. However, there are some remarkable exceptions: plants of INEDI and TEKNY genotypes are grouped according to treatment. Moreover, treated plants of both genotypes are clustered together, forming a clearly distinct group from the rest of individuals under FDS.

The classification under FIS provides a very different picture. Two main groups emerge, which split apart treated and control plants under this stress implementation strategy. The repartition of individuals in both clusters is, nevertheless, uneven. Indeed, all six individuals, whether treated or not, of SF193, SF107 and SF028 genotypes cluster together. Remarkably, SF193 control plants are grouped within the main cluster containing most of the treated plants of other genotypes, showing that, even when well-irrigated, SF193 displays gene expression levels similar to stressed plants. On the contrary, SF107 and SF028 plants were grouped within the main cluster containing most of the control individuals of the other genotypes. This indicates that control and drought-treated plants of those two genotypes are largely modulating their gene expression in the same way and that maybe these genotypes exhibit intrinsic differences in their response to the drought when compared to the other genotypes in our study.

### Differential analysis

Two independent ANOVAs were performed, one for each stress scenario implemented in our study. In order to handle false positives, Family Wise Error Rate (FWER) was corrected on the obtained p-values for each tested effect using Bonferroni's method [41]. Transcript expression levels producing corrected p-values <0.05 were considered significantly different. The results of the ANOVA, which are given in detail in Table S2, are summarized in Table 1.

**Table 1:** Genes showing ANOVA effects for two drought scenarios.

			FDS											
			All	g_o	g_t	g*t_g	g*t_o	g*t_t	t_o	Eff	t	g	g*t	None
		total	265	6009	300	197	31	12	102	6916	679	6771	505	25507
FIS	All effects (All)	15	3	6	1	2	0	0	2	14	6	12	5	1
	Only genotype effect (g_o)	4285	5	3239	29	40	1	0	4	3318	38	3313	46	967
	Genotype and Treament effects (g_t)	939	84	491	105	43	1	0	12	736	201	723	128	203
	Genotype and g*t effects (g*t_g)	12	0	7	0	2	0	0	0	9	0	9	2	3
	Only g*t effect (g*t_o)	18	0	0	0	0	0	0	0	0	0	0	0	18
	Treatment and g*t effects (g*t_t)	6	3	1	0	1	0	0	0	5	3	5	4	1
	Only treatment effect (t_o)	1701	163	354	126	78	16	12	67	816	368	721	269	885
	No effect (None)	25447	7	1911	39	31	13	0	17	2018	63	1988	51	23429
	At least one effect (Eff)	6976	258	4098	261	166	18	12	85	4898	616	4783	454	2078
	At least treatment effect (t)	2661	253	852	232	124	17	12	81	1571	578	1461	406	1090
	At least genotype effect (g)	5251	92	3743	135	87	2	0	18	4077	245	4057	181	1174
	At least g*t effect (g*t)	51	6	14	1	5	0	0	2	28	9	26	11	23

Counts of genes showing genotype, treatment and/or genotype*treatment interaction (g*t), based on the ANOVA analysis carried out with eight sunflower genotypes undergoing two drought stress scenarios in controlled environement: Fixed Duration Stress (FDS) and Fixed Intensity Stress (FIS).

**Differential analysis of plants subjected to Fixed Duration Stress.** The ANOVA on FDS plants revealed a total of 6 919 genes differentially expressed, out of which 6 771 displayed genotype-dependent expression profiles and 679 showed treatment-responsive modulation. Interestingly, 505 genes displayed significant expression modification under g*t interaction, meaning that those genes might be responsible for the different responses of each genotype to water deprivation. Tukey's test for Honest Significant Differences (HSD) revealed that 476 and 257 out of those 505 genes modulated under g*t interaction responded to treatment under FDS in INEDI and TEKNY plants, respectively (Table S2). On the contrary, very few genes produced treatment-related HSD in the other genotypes. This is in agreement with what was observed in the double classification, where INEDI and TEKNY were the only genotypes whose treated and untreated individuals emerged in separate clusters. This would imply that vigorous genotypes such as INEDI and TEKNY, would deplete available water more hastily because of their higher growth rate and bigger leaf surface and, therefore, would reach homeostasis-menacing soil water levels sooner than the other genotypes. Subsequently, gene expression regulation aimed at reacting to water scarcity would be implemented in these vigorous genotypes at an earlier date.

Gene Ontology enrichment tests were performed in order to unravel which Biological Processes were overrepresented in genes sharing the same effect(s) in the ANOVA (Table 2). Genes presenting treatment effect in FDS plants were particularly enriched in terms related to different responses to abiotic stress, including “response to water deprivation” (GO:0009414), and, interestingly, “response to abscisic acid” (GO:0009737). Genes whose expression responds to the g*t interaction under FDS were particularly enriched in similar terms. In contrast to this, genes whose expression under FDS is modulated according to the genotype were not enriched in any GO terms concerning stress or hormone responses.

**Table 2:** GO term enrichment test results for genes showing ANOVA effects for two drought scenarios.

	Effect	GO ID	GO term	Query item/total	Reference item/total	p-value FDR
FDS	At least treatment	GO:0050896	response to stimulus	131/497	1375/8415	4.60E-05
		GO:0006950	response to stress	85/497	787/8415	0.0001
		GO:0009414	response to water deprivation	20/497	80/8415	0.00025
		GO:0009415	response to water	20/497	85/8415	0.00034
		GO:0042221	response to chemical stimulus	78/497	736/8415	0.00034
		GO:0009628	response to abiotic stimulus	63/497	562/8415	0.00061
		GO:0009737	response to abscisic acid stimulus	20/497	127/8415	0.041
	At least genotype	GO:0044281	small molecule metabolic process	357/3373	659/8415	0.0085
	Only genotype	GO:0044281	small molecule metabolic process	319/3033	659/8415	0.029
	At least g*t	GO:0009414	response to water deprivation	15/368	80/8415	0.0046
		GO:0050896	response to stimulus	95/368	1375/8415	0.0046
		GO:0009415	response to water	15/368	85/8415	0.0046
		GO:0042221	response to chemical stimulus	58/368	736/8415	0.0046
		GO:0006950	response to stress	60/368	787/8415	0.0056
		GO:0009628	response to abiotic stimulus	47/368	562/8415	0.0056
		GO:0010035	response to inorganic substance	19/368	159/8415	0.026
	Only g*t	GO:0050896	response to stimulus	64/202	1375/8415	1.70E-05
		GO:0009414	response to water deprivation	14/202	80/8415	1.70E-05
		GO:0009415	response to water	14/202	85/8415	1.70E-05
		GO:0006950	response to stress	41/202	787/8415	0.00032
		GO:0009628	response to abiotic stimulus	33/202	562/8415	0.00032
		GO:0042221	response to chemical stimulus	39/202	736/8415	0.00032
	Genotype and treatment	GO:0006412	translation	25/237	302/8415	0.0029
		GO:0050896	response to stimulus	63/237	1375/8415	0.012
		GO:0006950	response to stress	42/237	787/8415	0.012
		GO:0044249	cellular biosynthetic process	61/237	1324/8415	0.012
		GO:0009058	biosynthetic process	64/237	1381/8415	0.012
		GO:0009628	response to abiotic stimulus	33/237	562/8415	0.012
		GO:0031408	oxylipin biosynthetic process	5/237	17/8415	0.037
		GO:0044283	small molecule biosynthetic process	20/237	292/8415	0.042
		GO:0031407	oxylipin metabolic process	5/237	19/8415	0.044
FIS	At least treatment	GO:0009628	response to abiotic stimulus	171/1687	562/8415	0.0043
		GO:0050896	response to stimulus	352/1687	1375/8415	0.013
		GO:0006950	response to stress	215/1687	787/8415	0.031
	Only treatment	GO:0009628	response to abiotic stimulus	118/1127	562/8415	0.021
	At least genotype	GO:0008152	metabolic process	1142/2710	3091/8415	0.0016
		GO:0044237	cellular metabolic process	954/2710	2573/8415	0.012
		GO:0044281	small molecule metabolic process	283/2710	659/8415	0.036
		GO:0009987	cellular process	1219/2710	3414/8415	0.042
	Only genotype	GO:0008152	metabolic process	926/2217	3091/8415	0.042
		GO:0044237	cellular metabolic process	781/2217	2573/8415	0.043
		GO:0009987	cellular process	1006/2217	3414/8415	0.043
	At least g*t	GO:0008152	metabolic process	311/677	3091/8415	0.0035
		GO:0009058	biosynthetic process	158/677	1381/8415	0.0063
		GO:0044281	small molecule metabolic process	87/677	659/8415	0.0088
		GO:0044283	small molecule biosynthetic process	46/677	292/8415	0.02
		GO:0044249	cellular biosynthetic process	148/677	1324/8415	0.02
		GO:0044237	cellular metabolic process	255/677	2573/8415	0.036

GO term enrichment tests performed on groups of genes showing genotype, treatment and/or genotype*treatment interaction (g*t) effects in ANOVAs carried out with eight sunflower genotypes undergoing two drought stress scenarios in controlled environment: Fixed Duration Stress (FDS) and Fixed Intensity Stress (FIS). Reference dataset corresponded to the GO terms available for the 32 423 sunflower clusters used for this work. Tests were performed on the AgriGO website [105].

Gene Ontology (GO) enrichment tests revealed that the 505 g*t-modulated genes were significantly enriched in “response to water deprivation” annotation. All the g*t-modulated genes with such GO annotation rendered significant HSD in at least one genotype, i.e. INEDI. Those genes included homolog sequences to well-known drought-responsive genes in Arabidopsis such as ABI2 (HuCL15555C001) and RD26 (HuCL01003C001). The ABI2 homolog was significantly upregulated in water-deprived TEKNY and, especially, INEDI individuals, with fold changes higher than 7 and 17, respectively. *ABI2* encodes a protein phosphatase 2C homolog to *ABI1* and it was primarily spotted because its mutation decreases ABA sensitivity. Both *ABI1* and *ABI2* transcripts have been shown to accumulate in response to ABA, suggesting a role of these two genes in a negative feedback mechanism, though at different levels, in the ABA-mediated signaling pathway [42]. Another gene homolog to AHG3/PP2CA (HuCL03720C001), which encodes a different phosphatase 2C presenting “response to water deprivation” annotation, is also g*t-modulated and thus significantly upregulated in treated plants of three genotypes under FDS, namely INEDI, TEKNY and SF109. It has been reported [43] that this gene may act as a negative regulator of the ABA signaling. However, some authors propose that its function might be limited to embryogenesis and early vegetative development, whereas others postulate that the gene might be involved in stomatal movement [44], [45]. More intriguingly, it has been shown that suppression of the expression of this gene accelerates cold acclimation [46]. Notwithstanding this, it had been previously reported that the closest homolog to PP2CA in tobacco enhances drought resistance [47].

A homolog to the Arabidopsis gene *LOX2* (HuCL00491C001), a chloroplast-localized lipoxygenase annotated as responsive to water deprivation, is significantly modulated under g*t in treated FDS individuals of INEDI and TEKNY genotypes. This gene has repeatedly been linked to wound-induced jasmonic acid accumulation [48], [49]. It has also been shown that it is sharply downregulated in jasmonate-mediated leaf senescence [50]. Moreover, it has been reported to be upregulated under slight drought stress and, on the contrary, repressed under stronger water deprivation [51]. The fact that in our study LOX2 appears upregulated reflects that our stress conditions correspond to a moderate stress in reference to that study where the authors considered drought stress as “severe” when RWC was lower than 48%. In our case, even though the FTSW was very low on these water-deprived INEDI and TEKNY individuals, they displayed RWC values around 80%. In addition, a homolog to the gene encoding the Phospholipase D Alpha 2 subunit or pldA (HuCL01497C001) is also significantly unregulated in our assay under the same conditions on the same genotypes. It has been reported that jasmonate concentration decreases in pldA-suppressed plants and that this effect is correlated with decreased levels of *LOX2* transcripts. It was thus proposed that LOX2 might be a downstream target of *pld* in mediating jasmonic acid accumulation [52].

A homolog to the Senescence Associated Gene *SAG21* (HuCL01066C004) is also upregulated in response to water deprivation in treated FDS plants of INEDI and TEKNY genotypes. Despite the fact that the actual role of this gene in plant senescence remains elusive, it was proposed as an early indicator of senescence whose expression peaks before the symptoms, such as leaf yellowing [53]. Moreover, it has been previously reported to be involved in drought- and nitrate-induced senescence in Arabidopsis [54], [55].

Another putatively nitrate-induced gene that is downregulated in water-deprived INEDI and TEKNY FDS plants is a homolog to nitrate transporter *NRT1.1* (HuCL02647C002). NRT1.1 has been proposed to be not only required in nitrogen uptake, but also a key player at the interface between nitrate and auxin signaling in plant development [56], [57]. Besides, the activity of NRT1.1 has also been associated in leaves to stomatal movement: nrt1.1 mutants presented lower stomatal conductance and higher adaptability to drought [58]. Downregulation of that gene in INEDI and TEKNY could imply an active response of these genotypes in order to close stomata under drought stress. However, the downregulation of NRT1.1 is more likely related to actual nitrogen uptake. This would be in agreement with the fact that a homolog to NRT1.2 (HuCL04010C001), a nitrate transporter not related to stomatal conductance, is upregulated in the same genotypes under FDS.

A sunflower homolog to RD26 (HuCL01003C001), a NAC transcription factor involved in a novel ABA-dependant signaling pathway in response to abiotic cues in Arabidopsis [59], was also significantly upregulated in treated INEDI and TEKNY FDS plants. In that study, the authors summarized different genes that were upregulated by RD26 in response to environmental stress. Sunflower homologs to two of these genes were significantly upregulated in our study in the same conditions as RD26 under FDS. These transcripts, namely HuCL00001C108 and HuCL01232C001, encode homolog proteins to Universal Stress Protein (USP; At3g62550) and lysine ketoglutarate reductase and saccharopine dehydrogenase (LKR/SDH; At4g33150), respectively. Even though the former was not described as drought-responsive by Fiujita and collaborators, a recent study on USPs has shown that, indeed, At3g62550 responds to water deprivation [60]. As for the LKR/SDH, it encodes a key enzyme in lysine catabolism and it has been reported to be upregulated in drought response in mandarin trees [61]. Lysine acts as carbon and nitrogen sink in the vacuole and its catabolism is increased under stress conditions by upregulating LKR/SDH. Lysine catabolism thus enhances amino acid to sugars conversion in sugar-starved plants. It also generates proline and pipeolic acid, two stress-related molecules, as well as other mediators in stress responses such as glutamate, nitric oxide and polyamines. Proline accumulation in response to drought and to ABA has been demonstrated in several species, including sunflower [62]–[64]. Proline accumulation also depends on the Δ1-pyrroline-5-carboxylate synthase (P5CS), which is upregulated under drought stress and whose suppression decreases drought tolerance, in Arabidopsis [65]. In our study, a homolog to P5CS1 (HuCL02382C003) is among the genes sharing GO term for “response to water deprivation” that are significantly upregulated in treated FDS individuals, namely of INEDI and SF109 genotypes. Moreover, another gene encoding a homolog to P5CS2 (HuCL02382C001), a protein sharing an overlapping role with P5CS1, is also significantly upregulated in INEDI, SF109, as well as TEKNY FDS, water-deprived plants.

Another gene showing g*t modulation under FDS and associated to GO:0009414 term corresponds to HuCL00842C001, an homolog to *Squalene Epoxidase 1* (*SQE1*, also known as XF1) . This gene encodes a key enzyme in the biosynthesis of sterols and its mutation has been proven to produce extreme drought hypersensitivity in Arabidopsis [66]. The authors in that study showed that sterols regulate Reactive Oxygen Species (ROS) through localization of RHD2 NADPH oxidase. Thus, defective handling of that enzyme in the sqe1 mutant would be responsible for the hypersensitive drought response.

More intriguing are the results obtained for a homolog to *AVP1* (HuCL06154C001), a gene encoding a vacuolar H+ Pirophosphatase whose overexpression has been associated with drought tolerance in Arabidopsis and tomato [67], [68]. In fact, one sunflower homolog to this gene in our study seems to be significantly down regulated in INEDI FDS plants under water deprivation (FC∼−3.5). It must be pointed out that AVP1 has also been proposed to hamper cell division in auxin-mediated organogenesis. Thus, we may speculate that the downregulation of AVP1 in INEDI would be related to leaf surface reduction in response to water deprivation and/or drought-related detoxification.

**Differential analysis of plants submitted to Fixed Intensity Stress.** The number of gene modulated at least under one factor in FIS plants, that is 6 976, was very similar to the total of 6 916 genes transcriptionally regulated in FDS plants. However, even though 4 898 genes were modulated in both FDS and FIS situations, the relevance of each factor in either stress implementation strategy was very different as detailed in Table 1.

Genes showing a g*t modulation of their expression under FIS are worth closer attention because they could, by definition, support genotypic differences to the same water constraint in our drought tolerant and sensitive genotypes. Fifty-one genes showed a genotype*treatment interaction effect under FIS and 11 of them showed the same effect under FDS. It is worth pointing out that among those 11 genes, at least three of them are putatively involved in cell wall modifications. Notably, there is an homolog to the *β-1,3-*glucanase *BG1* (HuCL04869C001). *BG1* was previously shown to be downregulated under drought stress in Thellungiella, a close relative of Arabidopsis. This species grows in harsh environments and has been used as model organism in transcriptomic studies on abiotic stress, including drought [69], [70]. BG1 significantly reacts under both stress implementation scenarios in our study, presenting all genotype, treatment and g*t effects. Tukey's test revealed significant treatment-depending HSD values in 3 genotypes in both FDS an FIS, namely INEDI, SF193 and TEKNY. Besides, HSD values are also significant for SF109 under FDS and SF107 under FIS. Even though the role of BG1 under drought stress remains unknown, our findings underline the importance of cell wall modifications in genotype-dependent responses to drought in sunflower. Moreover, among the 11 genes rendering g*t modulation under both stress implementation strategies, we found also homolog genes to At1g23200 (HuCL02872C001), which encodes a pectin esterase, and to TET3 or TETRASPANIN3 (HuCL02666C001), a senescence-related protein. Both TET3 and pectinesterases have been reported to be involved in arabinogalactan-derived cell to cell signaling at the cell wall level [71].

As opposed to what was observed under FDS, however, genes whose expression responded to the g*t interaction under FIS were not enriched in terms involving abiotic stress responses. This might be due to the much reduced number of genes modulated under g*t interaction in FIS plants as compared to FDS individuals. Among those 51 genes, we could find genes well known to be involved in drought responses and subsequent biological processes such as, for example, redox mechanisms and cell wall rearrangements. Furthermore, we found genes encoding proteins that have been reported to be altered in ABA-mediated stress responses, other than the already mentioned BG1. This is the case, for instance, of the cell wall-related glycosyl hydrolase BGLU16 (HuBU032078), which has been shown to be up-regulated by ABA but repressed by drought [72]. Another gene that might be modulated by ABA is a homolog to the Arabidopsis ALDH10A9 (HuCL00113C001), which encodes an ABA-responsive aldehyde dehydrogenase that has been shown to be targeted to peroxisomes, being involved in detoxifying aminoaldehydes produced under stress [73]. That study confirmed the hypothesis that this enzyme is involved in the oxidation of aminoaldehydes resulting from the activity of the copper amine oxidase (CAO; At2g42490) and the pheohorbide A oxygenase (PAO or ACD1; At3g44880) in the peroxisomes. Interestingly, a homolog to CAO (HuCL06038C001) is also a member of the 51 genes whose expression is modulated under g*t interaction in FIS individuals. This reveals the importance of aminoaldehyde detoxification in the genotype-dependant responses in sunflower to harsh water deprivation. Among those 51 genes there are homologs to other genes putatively involved in redox mechanisms. That is the case of PRXR1(HuCL00049C001), ATFRO7 (HuCL12107C001), the Glucose-methanol-choline (GMC) oxidoreductase AT1G73050 (HuCL04787C001), and also cytochrome P450 enzyme *CYP82C* (HuCL02115C001), which modulates jasmonate-induced root growth inhibition and defense gene expression [74]. Indeed, jasmonic acid plays very important roles in response to biotic cues. However, its involvement in abiotic stresses and, more particularly drought, remains elusive. This is likely due to the fact that its interaction with ABA presents synergistic and antagonistic elements [72]. There is another jasmonate-responding gene (i.e. AT4G08870), among those modulated by g*t interaction in FIS plants (HuCL00001C196). This gene encodes an arginase that has been proven to be preferentially expressed in the leaves, and has been shown to be involved in MYC2-mediated resistance to insects [75]–[77]. This gene had already been proven to be jasmonate-responsive in another study, where the authors proposed that the coordinated activation of metabolic pathways for antioxidants and defense compounds by jasmonate provides stress tolerance in Arabidopsis. Another gene that has been shown to be wound- and jasmonic-responsive is PTR2, which encodes a member of the Major Facilitator protein superfamily [78]. A sunflower homolog to PTR2 (HuCL14745C001) is also among the g*t-altered genes in FIS plants. Finally, it should be pointed out the presence of an homolog to the Arabidopsis aquaporin PIP2;5 (HuCD846314). Transcriptional variations of this gene, along with that of other aquaporins, have been shown to be to be linked to leaf water content.

Drought treatment affected 505 genes under FIS. As a matter of fact, 406 out of them showed g*t effect under FDS indicating that g*t effect under FDS mixes treatment and true g*t interaction through the effect of the genotypic growth differences on the water consumption resulting in different constraint intensities. Moreover, 269 out of those 406 genes were only altered by treatment. This implies that the expression of those genes is only regulated under severe water scarcity. Hence, their transcription would not be altered in FDS plants of genotypes not having attained significantly reduced ITW levels (see Table 1). Correspondingly, the number of genes regulated upon treatment in FIS plants (i.e. 2 661) was much higher than in FDS plants (i.e. 679 genes). Gene Ontology studies revealed that genes presenting treatment effect in FIS plants were enriched in terms involving stress responses, most particularly to abiotic stimulus.

On the opposite and similarly to what happened under FDS, genes showing a genotype effect under FIS were not enriched in these terms.

### Covariance between transcriptomic data and morpho-physiological variables

**Plants under FDS.** In order to join together the transcriptomic and the physiological data, Sparse Partial Least Squares (SPLS) analysis were conducted using the mixOmics [R] package [35], [79]. SPLS was especially conceived to deal with high dimensional data sets and, more particularly, with experimental designs where the number of variables (genes and physiological variables combined) exceeds the number of samples to be considered. The SPLS produces not only stable variable comparisons but it also allows highly valuable variable selection, which made it highly suitable for our study [80].

**Figure 3:**
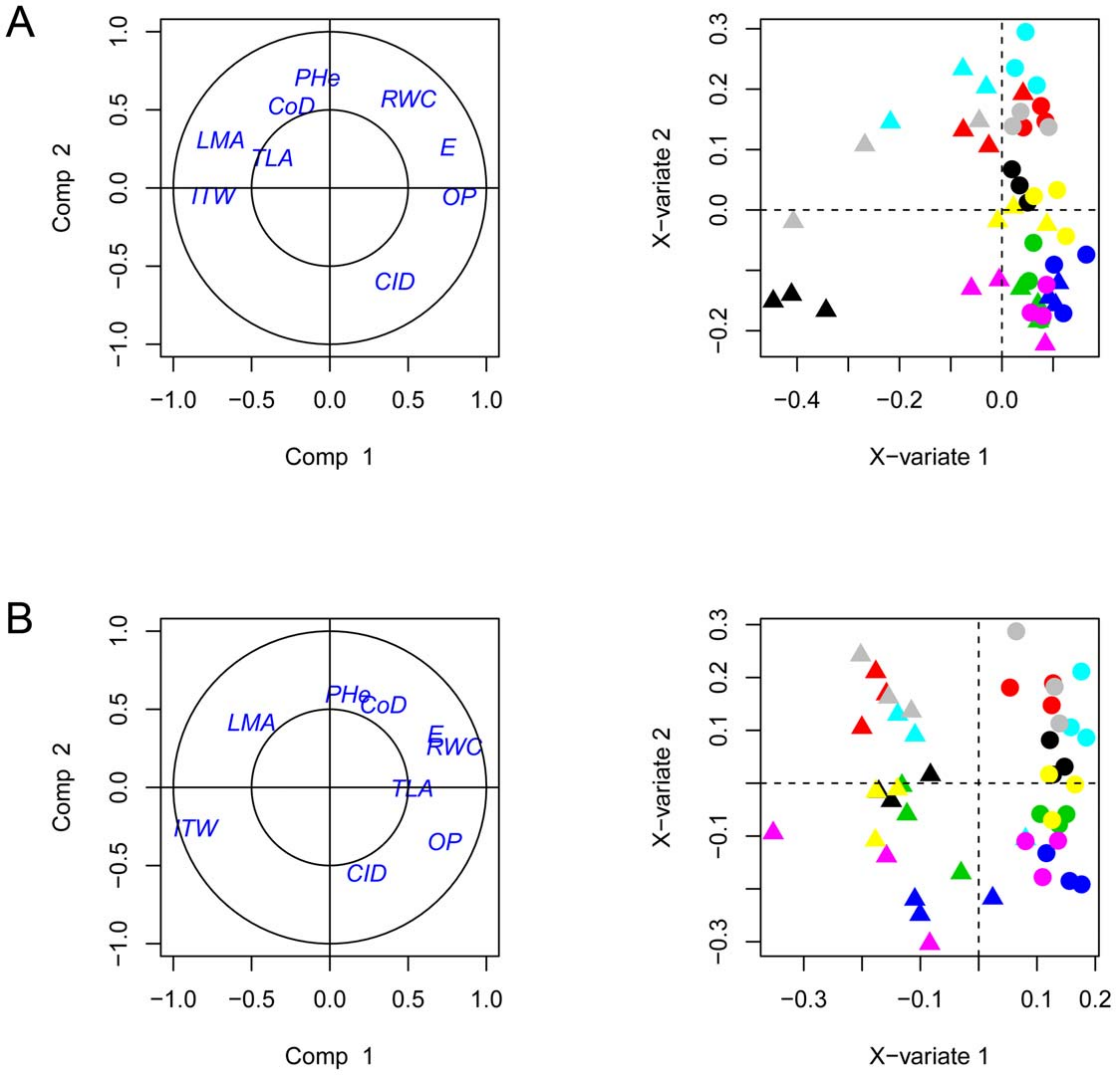
Results of the sparse Partial Least Squares (SPLS) analysis on plants under FDS (A) and FIS (B). Plots show respectively the repartition of the morphophysiological variables (left) and individuals (right) along the first two components of the SPLS. Morphophysiological variables are carbon isotope discrimination (CID), collar diameter (CoD), Transpiration rate (E), Integrated Transpired Water (ITW), Leaf Mass per Area (LMA), Osmotic Potential (OP), Plant Height (PHe), Relative Water Content (RWC) and Total plant Leaf Area (TLA). Triangles correspond to treated plants whereas circles correspond to their untreated counterparts. Genotypes are color-coded as follows: Inedi (black), Tekny (gray), Melody (red), SF109 (turquoise), SF326 (yellow), SF193 (magenta), SF028 (green) and SF107 (blue).

One SPLS analysis was carried out for each stress scenario The first feature that comes up under FDS is that ITW and Osmotic Potential (OP) clearly define the first axis of the SPLS and that they are negatively correlated (see Fig. 3). In other words, this first axis accounts for the expected negative correlation between the faster water depletion and a lower OP, i.e. with a stronger osmotic adjustment. Leaf Mass per Area (LMA), expressing the dry mass per area unit on the reporter leaf (in g/m^2^), appears also found negatively correlated with OP, exhibiting the fact that plants with reduced cell growth on the leaves would present stronger osmotic adjustment. The profiles of treated plants, and most particularly those of INEDI, TEKNY and SF109 genotypes, are associated with high ITW and strong osmotic adjustment, whereas control plants are associated with low ITW and reduced osmotic adjustment. This result indicates that INEDI, TEKNY and SF109 would lower their OP in response to severe water scarcity, which would allow maintaining cell turgor. Because their ITW values were lower at harvest, treated plants of the other genotypes were less confronted to cell water loss and could thus keep up with water homeostasis without turning to osmolyte accumulation.

ITW and the Total Leaf Area (TLA), the variable that sums up the surface of all leaves in the plant, are, to some extent, positively correlated. This can be explained by the fact that genotypes with higher growth rate, produced higher TLA before stress application. Hence, once treatment was implemented, those plants underwent steeper water depletion due to higher transpiration. However, because the correlation between both variables is not strong, we may speculate that for equivalent TLA, every genotype did not consume the same amount of water and therefore have different water stomatal and/or non-stomatal conductance. Furthermore, ITW is likewise correlated to LMA. The observed high LMA values are correlated to smaller cells and reduced cell expansion. Decreasing cell expansion in response to drought is a well-described long-term strategy in order to reduce water transpiration, and it is one of the genetic parameters taken in by the sunflower crop model SUNFLO [81]. In the FDS part of our assay, this is especially true for INEDI and TEKNY, two hybrids with large leaf areas and consequently with high ITW at harvest time.

Another response plants may implement in order to keep up with water homeostasis is reducing stomatal conductance. Under FDS, ITW is negatively correlated with transpiration rate (E). This negative correlation implies that genotypes undergoing abrupt water depletion will reduce stomatal conductance in order to avoid severe tissue water loss, hence the lower Rvalues. This is in agreement with the fact that treated INEDI and TEKNY FDS individuals show a tendency towards lower Carbon Isotope Discrimination (CID) values than their irrigated counterparts, giving us an indication of higher Water Use Efficiency (WUE) [82]. This is also true, to a lesser extent, for other genotypes, such as SF109 and MELODY (Fig. S2). Hence, in the light of our results, plants from every genotype undergoing low enough FTSW values would tend to close the stomata more progressively and lower down their CID. This would imply an increase of WUE but of less importance than for TEKNY and INEDY with, eventually, lower productivity.

Moreover, it is worth pointing out the fact that, on the second axe of the SPLS concerning plants under FDS, CID appears in opposition to morpho-physiological features such as Plant Height (PHe) and Collar Diameter (CoD). We may thus speculate that, independently of FTSW, the most vigorous genotypes made a better use of the available water. Therefore, plants undergoing lower stomatal conductance and subsequent lower carbon intake would be thinner and smaller.

Overall, it can be observed that plants under FDS are discriminated along the first axis of our SPLS according to water depletion and subsequent osmotic adjustment. Genotypes with a leaf surface large enough (e.g. hybrids such as INEDI and TEKNY) would be the first suffering drought and implementing transpiration rate reduction and osmotic adjustment. On the contrary, plants are discriminated on the second and third axes according to their genotype, with little regard to lack of water.

As it happened to CID, Relative Water Content (RWC) also appears between both components 1 and 2, displaying positive correlation with PHe and CoD as well as with CID, E and OP. Therefore, in our conditions on the studied genotypes, that plants with higher RWC values were more vigorous and they were less prompt to head towards osmotic adjustment. In fact, plants would implement osmolyte accumulation in response to water deprivation in order to be able to keep up with cell turgor even in the case of cell water loss, reflected by lower RWC values. In this sense, it is worth noting that RWC appears, to some extent, in negative correlation to LMA. Because lower LMA values imply larger cells and, hence, stronger cell expansion, we may argue that higher RWC values favor turgidity and hence cell expansion in accordance with previous results [17], [83]

**Plants under FIS.** Plants under FIS where harvested at different dates when their FTSW values were below 0.1 and close to an average of 0.04 (as opposed to FDS, where all plants were harvested on the same day, therefore producing different FTSW values). In spite of these divergent stress implementation scenarios, the measured morpho-physiological variables do place themselves in the SPLS analysis not very differently as they do under FDS. However, there is one noticeable exception. Under FIS, ITW appears negatively correlated to TLA, as opposed to what happened under FDS, where they were positively correlated. Indeed, it should be kept in mind that, as we have stated above, ITW under either stress scenario has different implications. In the case of FIS stress implementation, higher TLA values provoke that plants reach an FTSW value close to 0 at an earlier date than plants with lower TLA, because higher TLA means higher evapo-transpiration. Because TLA is placed differently under FIS than under FDS it is also tempting to speculate that, in agreement to that, plants under FIS endured strong and long enough a drought stress so that their adaptative strategies are easier to track down. Thus, it can be observed that, in fact, TLA is negatively correlated to LMA.

If we focus on how FIS individuals are placed in this SPLS analysis, we realize that the first component is neatly driven by the “treatment” effect. That is, irrigated and water-deprived individuals of all genotypes locate themselves at similar coordinates along the first axis. As it happened under FDS, components 2 and 3 of the SPLS under FIS managed as well to differentiate genotypes, being PHe, CoD and CID their main driving variables. LMA, E and OP variations are captured by both components 1 and 2, being thus driven by both treatment and genotype. These physiological traits constitute therefore important indicators to describe genotype-specific drought responses.

### Gene-Phenotype Networks relating gene expression and morpho-physiological variables

The above-mentioned SPLS regression analysis allowed us to infer networks displaying relevant relationships between morpho-physiological variables and gene expression under FDS and/or FIS. SPLS combines a multivariate projection-based method comprising a lasso penalization-mediated variable selection. Associations are then inferred by means of pairwise association scores between variables from both data frames containing gene expression and morpho-physiological data.

**Gene-Phenotype Network in Fixed Duration Stress scenario.** In the case of plants under FDS, a total of 690 genes displayed absolute association scores higher than 0.65 with at least one morpho-physiological variable, producing a total of 1 236 associations, 579 being positive and 657 negative correlations (Fig. S3). No gene was associated at that threshold with CoD or TLA, six were linked to PHe and there were 38 genes whose expression was correlated to LMA values. Tighter correlation with gene transcription was observed for E (388 genes), ITW (208 genes) and, most particularly, OP (576 genes). In the case of OP, nearly half of those 576 genes, namely 256, were associated exclusively to this variable. Remarkably, all 208 genes related to ITW were also linked to, at least, OP. Moreover, 189 out of those 208 genes were correlated with E, albeit, as it happened with OP, in the opposite sense to ITW. It is worth pointing out that no gene was associated exclusively with ITW (i. e. FTSW in FDS scenario), implying that gene expression was not correlated exclusively with the available water for the plant. The fact that this variable appears in combination with other variables might reflect that the water constraint (captured by ITW) will trigger plant responses which will then have an impact on the evolution of water consumption and consequently on ITW itself. Thus, the two main morpho-physiological variables correlated with gene expression are OP and E, which will thus have an impact on ITW. Gene ontology enrichment tests on those 208 genes related at least to ITW and OP, and in most cases to E as well, revealed an enrichment in “Response to abscisic acid stimulus” (GO:009737) term, suggesting the major role of ABA in progressive drought stress response. Likewise, molecular functions concerning “symporter activity” (GO:0015293) as well as “transmembrane sugar transporter” (GO:0051119) and “water channel activity” (GO:0015250) were significantly over-represented, underlining the importance of osmotic adjustment in the implemented stress.

**Gene-Phenotype Network in Fixed Intensity Stress scenario.** For plants subjected to FIS, a total of 1 032 genes produced associations with a score higher than 0.65 with at least one morpho-physiological variable, rendering a total of 1 967 associations, of which 1 026 were positive and 941 negative correlations (Fig. S4). Nearly half of those 1 032 genes, i.e. 459, appeared also on the FDS network. As it happened with FDS plants, no gene was associated with CoD or TLA. Conversely to what happened under FDS, though, no gene was correlated to LMA. Likewise, whereas only 6 genes were linked to PHe under FDS, a total of 24 were so in FIS plants. More strikingly, OP is not the variable associated with highest number of genes under FIS. In fact, under FIS, the expression of 176, 337 and 514 genes were linked to, respectively, OP, Rand RWC. Nonetheless, the variable that produced more associations was ITW, which was correlated to the expression of 916 genes. However, as opposed to associations under FDS, where no gene was exclusively related to ITW, 252 genes were so under FIS. The expression of 197 genes out of those 252, that is 78%, do not produce any association with morpho-physiological variables in plants under FDS. The expression levels of these genes are thus exclusively correlated with the time individuals take to reach harsh stress levels, which is captured by ITW under this stress implementation. Gene ontology enrichment tests did not reveal any significant GO term abundance.

**Figure 4:**
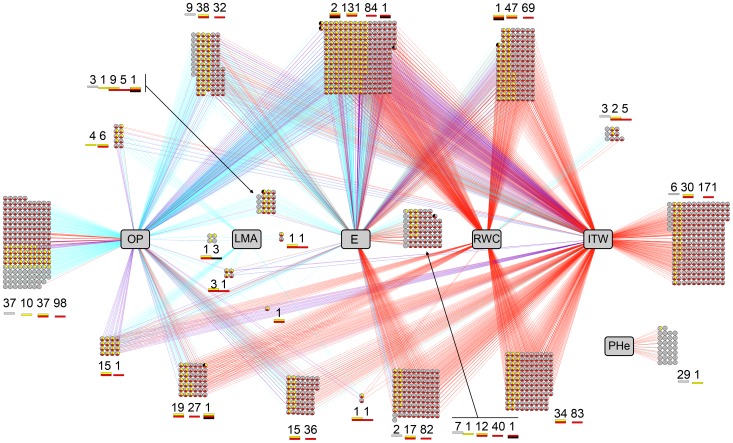
Gene-Phenotype network produced by SPLS, based on responses of eight sunflower genotypes to two drought stress scenarios implemented in controlled environment. Genes presenting absolute correlation scores higher than 0.65 with at least one morpho-physiological variable are represented. Each circle represents one gene. Blue, red and purple edges indicate, respectively, whether the gene-phenotype association exists under FDS, FIS or both stress scenarios. Each gene circle is split in three slices displaying ANOVA results. Yellow, red and black slices represent, respectively, treatment effect under FDS (moderate stress responsive genes), treatment effect under FIS (severe stress responsive genes), and g*t effect under FIS (gene likely to explain genotypic differences in stress responses). Numbers of genes for each combination of ANOVA effects are shown for each gene-phenotype group. Phenotypic responses are in gray squares, OP: Osmotic Potential, LMA: Leaf Mass Area, E: Transpiration Rate, RWC: Relative Water Content, ITW: Integrated Transpired Water, PHe: Plant Height.

**Gene-Phenotype Integrated network.** The correlation networks built under FDS (Fig. S3) and FIS (Fig. S4) were merged into one unique network shown in Figure 4. This integration allowed us to display simultaneously not only genes exclusive to either FDS or FIS networks but also genes rendering associations under both stress implementations, though not necessarily with the same morpho-physiological variables in each case (see Fig. 4). Thus, among the 1 263 genes linked to at least one morpho physiological variable under FDS and/or FIS, there are 231 and 573 genes that appear exclusively in the FDS or the FIS networks, respectively. On the other hand, 459 genes are related to morpho-physiological variables under both stress implementation strategies. The fact that fewer genes appear exclusively under FDS might be because fewer genotypes were substantially altered under this stress implementation. However, links between gene expression and morpho-physiological variables under FDS might translate earlier responses to water deprivation. A total of 191 genes producing links with morpho-physiological variables exclusively under FDS are related to OP, that is 83% of the 231 genes. Moreover, 138 of those 191 genes are uniquely associated to OP, that is 60% of all the genes whose expression levels are correlated with morpho-physiological features under FDS (Table S3). This indicates that the adjustment of the osmotic potential in order to cope with eventual water loss while maintaining cell turgidity is an early response in sunflower under drought stress. Furthermore, the pre-emptive nature of this response is underlined by the fact that only eight genes are related to RWC. However, it is worth pointing out that all those eight genes were associated uniquely to RWC, and not to any other morpho-physiological feature. One of those eight genes, i.e. HuCL00871C003, is a homolog to the Arabidopsis *CAX1* gene, which encodes a Ca2+/H+-antiporter that has been shown to be crucial in uptaking apoplastic calcium by the mesophyll cells. CAX1 deficiency results in reduced cell wall extensibility, stomatal aperture, transpiration, CO2 assimilation and leaf growth, thus reducing plant productivity [84]. In the future, it might be worth studying in detail this link between RWC and CAX1 with regard to crop yield in sunflower.

Among the genes showing significant associations exclusively under FDS, 34 of them are related to ITW, though none of them is uniquely related to this variable. In fact, all those genes are also related to, at least, OP, while 27 of them (i.e. 79%) are associated with E as well. Our data shows that sunflower implements both the osmotic adjustment and the reduction of transpiration rate at the early stages of drought stress. Among those 34 genes, we found HuCL00001C110, a homolog to *RACK1* (Receptor for Activated C Kinase 1), whose encoded protein has been reported to be a critical negative regulator of ABA responses under abiotic stress. It has been proposed that this protein plays its cellular role by regulating protein translation, and that it may be required for normal production of 60S and 80S ribosomes [85], [86]. Indeed, “Translation” is the GO term particularly enriched in the ITW-related subset of genes: 12 out of 34 were associated with this GO term. A total of 11 genes in this group encode ribosomal proteins involved in the biosynthesis of 60S and 40S ribosomes. Furthermore, we find homologs to XERICO (HuCL05555C002) and LTP3 (HuCL00012C003). XERICO is a RING zinc-finger transcription factor involved in ABA homeostasis, presumably by activating the NEC3 gene and/or sending negative regulators of the ABA biosynthesis towards the ubiquitination pathway [87], [88]. LTP3, on the other hand, is an ABA-responding gene as well, involved in cell wall mobilization and cuticle thickening in response to biotic and drought stress [89], [90]. Because ITW reflects water availability under FDS, these FDS-exclusive genes linked to ITW might reveal constitutive expression patterns under water deprivation. That is, genes whose expression profiles are altered when drought is perceived henceforth remaining unmodified as long as such stress is in place.

Apart from ITW, all morpho-physiological variables in the merged network present associations with genes both under FDS and FIS stress implementations. However, only OP presents a group of 16 genes that are exclusively related to this variable both under FDS and under FIS. It is worth pointing out that all those genes but one encode proteins that are expected to be located either in the plasma membrane, in the vacuole or in the chloroplast. One of those genes, namely HuCL04973C001, is a homolog to the Arabidopsis gene *ITN1* (*Increased Tolerance to NaCl 1*), whose expression is positively correlated with the osmotic potential, that is negatively so with the osmotic adjustment. This gene encodes a member of the ankyrin repeat family that has been reported to positively regulate the production of Reactive Oxygen Species (ROS) in response to ABA under salt stress. However, it has been suggested that it might not be involved in ROS production under drought or osmotic stresses. Furthermore, it has been proposed that ITN1 is neither involved in ABA-mediated stomatal closure, where ROS act as secondary messengers [91]. Thus, the exact role of ITN1 under drought stress remains undefined. Our data suggest that ITN1 might be involved in osmotic adjustment in response to water deprivation. Interestingly enough, another gene in that shortlist of 16 is a homolog to EX1 or EXECUTER1 (HuCL02634C001), which encodes a plastid-located protein involved in singlet oxygen-induced upregulation of nuclear gene expression in response to environmental stress. However, EXECUTER1 seems to be integrated in a very complex stress-responsive signaling network that might be the subject to the control of various modulators, thus mitigating the harsh consequences of network partial dysfunction. It is worth pointing out that the expression of this gene in negatively correlated with the osmotic potential and, therefore, positively correlated with the osmotic adjustment [92], [93]. These results reveal links between ROS homeostasis and osmotic adjustment in response to drought stress that deserve further research. Indeed, it has already been suggested that under salt stress, compatible solutes usually involved in osmotic adjustment (e.g. glycine betaine, proline, mannitol, trehalose or myo-inositol) significantly reduce OH•-induced cellular K+ efflux and subsequent damage to membrane transporters. Most interestingly, this cell protective role was achieved also by solutes without any scavenging properties. Hence, it remains unclear whether the mitigation of oxidative damage by compatible solutes is the result of direct protection of membrane transporters or free-radical scavenging properties [94], [95].

### Differential gene expression and Gene-Phenotype Network

Most of the genes linked to one or more morpho-physiological variables present at least one differential effect under the ANOVA. In the case of FDS plants, the expression of 514 out of those 690 genes related to at least one morpho-physiological variable, was altered by treatment, genotype and/or the g*t interaction, and the response of 182 genes was found affected by both genotype and treatment. To great extent, these genes were related, at least, to E. Indeed, whereas 388 out of the 690 genes on the network, i.e. 56.2%, were at least related to E, a total of out 148 of the 182 genes (81%) displaying all differential effects, were related to E. As a general rule, the more the expression of a gene was associated with different variables, the higher the probability was for this gene to display all differential effects. Thus, if we look at the 16 genes related to all four LMA, OP, ITW and E, the expression of 11 of them is modulated under all three effects. This is also the case for 87 out of the 182 genes (48%), associated with all three OP, ITW and E. On the other hand, 176 genes out of the 690 genes related to at least one morpho-physiological variable do not present any significant modulation in their expression. Most of these genes, i.e. 103, appear associated exclusively to OP, representing 58.5% of the genes in the network whose expression is not differentially regulated. Interestingly, however, the expression of 147 out of those 176 genes is altered under FIS. The fact that they are not differentially expressed under FDS may be due to the fact that the stress perceived by certain genotypes at harvest was not enough to modulate their gene expression.

In the case of plants under FIS, 810 out of the 1 032 genes related to at least one morpho-physiological variable (78.5%), are modulated only by the treatment. Another 179 genes (17.3%) are modulated both by genotype and treatment. Only four genes were regulated by the treatment and the g*t interaction, whereas only one gene appeared altered by all three studied effects. Interestingly, 23 genes displayed only the genotype effect and all of them were exclusively related to plant height (PHe). Indeed, PHe appeared related to genes modulated uniquely by the genotype. In sunflower affected by a long and severe drought stress in the field, a reduction of the plant height can be observed. Our results might indicate that, in the implemented drought stress scenario, other morphological traits were affected before plant height. This was the case both under FDS and FIS. Notwithstanding this, it should be noted that the genes that intervened with PHe under FDS are different from the ones under FIS.

Unlike to what happened under FDS, 252 genes were exclusively related to ITW under FIS. The vast majority of those genes (197, i.e. 78%), were not associated with any morpho-physiological variable whatsoever under FDS. This was also the case for the 39 genes linked exclusively to E, 37 of which are not connected to any morpho-physiological feature under FDS. This is also the case for 89 out of the 117 genes relating to both ITW, that is 76%.

### Sunflower responses to drought in the field environment

The hybrid MELODY used in the greenhouse conditions was chosen to assess drought response of sunflower in the field. A total of 156 genes were differentially expressed between irrigated and non-irrigated MELODY individuals in the field. GO enrichment tests on those genes produced overrepresented terms concerning cellular amino acid metabolic processes. This might indeed reveal osmotic adjustment mechanisms were amino acids may be involved, as observed in the greenhouse experiment.

Among them, 84 (i.e. 54%) were modulated by treatment and/or g*t interaction in the greenhouse experiment, seven of them under FDS, 28 under FIS and 49 under both stress implementation strategies. This subset of 84 genes constitutes a robust and valuable group of candidate genes in order to assess sunflower drought stress in a wider range of environments.

**Figure 5:**
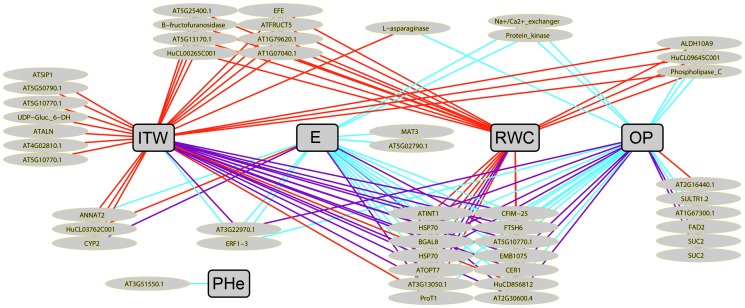
Gene-Phenotype sub-network produced by SPLS, based on responses of eight sunflower genotypes to two drought stress scenarios implemented in controlled environment. Only genes regulated by drought stress in field conditions are shown. Each ellipse represents one gene. Blue, red and purple edges indicate, respectively, whether the gene-phenotype association exists under FDS, FIS or both stress scenarios. Sunflower Heliagene cluster IDs are shown when meaningful names of Arabidopsis homologs are not available. Gray squares represent phenotypic responses; OP: Osmotic Potential, E: Transpiration Rate, RWC: Relative Water Content, ITW: Integrated Transpired Water, PHe: Plant Height.

Furthermore, we found 49 out of the 156 drought-regulated genes in the field environment (i.e. 31.4%) to be linked to phenotypes in the Gene-Phenotype network, thus underlining the physiological processes involved in drought stress response in our field experiment as shown in Fig. 5. GO enrichment tests highlighted a limited amount of Molecular Function terms on those 49 genes, including “Ion transmembrane transporter activity” (GO:0015075) and “Active transmembrane transporter activity” (GO:0022857) (see Table S4). Moreover, according to the ANOVA, the expression of all those 49 genes was treatment-altered under FIS and 41 showed a treatment and/or g*t interaction under FDS.

The presence among those genes of homologs to MAT3 (HuCL03862C001) and EFE (HuCL00039C002), two ethylene-related genes, reveal the key role of this hormone in drought stress response. *MAT3* encodes an S-adenosyl transferase involved in ethylene biosynthesis . The expression of *MAT3*, and therefore ethylene biosynthesis, has been reported to be drastically diminished in Arabidopsis plants expressing *HAHB-4*, a sunflower HD-Zip transcription factor transcriptionally regulated by water availability and abscisic acid [96]. Remarkably, EFE (Ethylene Forming Enzyme) has been previously reported to be repressed under drought stress in response to at least three hormones, including ABA [72]. In that work, authors highlighted the cross talk between the different environmental cues as well as among the subsequent hormone signaling pathways.

Over-representation of field drought regulated genes linked to a given phenotypic traits in the gene-phenotype network reflects the importance of this traits in this natural environment. This is the case for RWC-related genes showing a significant enrichment in the field dataset (28/49 vs 523/1263, p = 0.009) and possibly for genes linked to OP (28/49 vs 628/1263, p = 0.066). This approach based first on generating a general model of stress response (the gene-phenotype network), and secondly on testing the specific enrichment in genes linked to a phenotypic trait in an independent dataset is novel and allows us to clearly highlight the importance of RWC and OP physiological responses in field conditions.

## Conclusion

Because water constitutes more than 95% of some plant tissues, water deprivation might affect any molecular and physiological process. To improve our understanding of such a complex response, it is essential to develop systemic approaches to understand how the functional system is controlled by multiple factors. Furthermore, this approach can play a role in developing knowledge in less tractable experimental models and driving hypothesis for functional genomics studies.

In this work, we developed a dual drought scenario strategy and exploited genetic diversity in sunflower to decipher the molecular basis of drought responses and reveal physiologically relevant processes. Genotypic differences in the response to drought stress were very important but still a large number of genes were modulated by this treatment in controlled conditions. In order to relate gene expression to phenotypic variations, we inferred a gene-phenotype network. Major drought responses (E, RWC, OP, SLA, PHe) and stress intensity (ITW) could be statistically associated to gene expression modification. These results allowed us not only to confirm data previously obtained on model species (reviewed by [20] as well as smaller-scale data obtained on sunflower [97], [98], but also to open new doors to the study of drought responses in this species. Besides, this analysis identified key genes associated to one or several process whose expression regulation differs in sensitive and tolerant genotypes providing good candidates for further functional and genetic studies.

Few other studies in microorganism model systems, studying together physiological parameters and gene expression, were performed to link mainly metabolomic and transcriptomic datasets [99], [100]. They used various stresses to infer associations between physiological and gene expression variations. However, none exploited the genetic variability existing in these microorganisms.

In our study, the general drought Gene-Phenotype network inferred from data obtained in controlled conditions was used in an independent field experiment where physiological indicators were not tractable. The set of drought regulated genes in field overlapped significantly with controlled condition data. As a result, we could identify robust sunflower genes responding to drought in agronomical conditions and we could also assess the importance of the osmotic adjustment and the regulation of relative water content in sunflower in such environment.

Taken together, our work provides a new and large-scale expression dataset in sunflower undergoing drought stress, that is an important yield limiting factor for this crop in the frame of the climate change. We inferred statistically significant associations between several thousands of genes and phenotypic responses and modeled their overall interactions in a gene-phenotype network. More importantly, we were able to identify genes and physiological processes that could explain genotypic differences of drought responses in controlled and agronomic conditions.

## Materials and Methods

### Choice of genotypes

Eight sunflower genotypes (5 inbred lines and 3 F1 hybrids) were chosen for this study, paying attention to previous phenotyping data that provided evidence of genotype-dependent responses to different environmental cues, including water deprivation. SF193 and SF326 are two reference lines in our group as well as the parental lines of the INEDI RIL population developed by INRA. SF193 is a maintainer line whose pedigree includes the Progress cultivar, which improves the tolerance to Phomopsis and the resistance to Downy mildew, and the widely used HA89. Both SF193 and SF326 behaved differently in response to water deprivation in preliminary studies (Rengel *et al*, unpublished results). For instance, it was observed that SF193 closes its stomata at much higher soil water content than SF326 at the same developmental stage. INEDI, another genotype used in this work, corresponds to the F1 hybrid SF193*SF326. SF109, (also known as 2603) is an INRA-bred line that, despite its susceptibility to some diseases like Phomopsis, has been widely used as a female parental line in hybrid crossings in Spain and other Southern European countries due to its good agronomic adaptation to dry conditions. Two other genotypes, SF028 and SF107, have previously been used as male parental lines in field test-crosses in different locations, and both show highly contrasted yields between irrigated and non-irrigated conditions, depending on the location. Finally, TEKNY and MELODY are widely cultivated sunflower hybrids.

### Experimental design

**Growing conditions.** Plants were grown in the greenhouse and the experiment was conducted in May and June 2009. Greenhouse air temperature was kept at 17°C during the night and between 20 and 25°C during daylight.

Ninety six individual pots were arranged in six blocks,, each pot containing one single plant, to a final stand density of 6 plants/m^2^. Pots (25 cm diameter, 30 cm height) were filled with 15 liters of substrate (50% clay loam, 10% sand and 40% potting soil).

Each block contained two plants of each genotypes (2*8 genotypes). One of those two plants was used as a control (well-watered individual) and the other as a treated plant (water-deprived individual). Pots within each block were randomly arranged within a block. All pots were daily irrigated before the beginning of water treatments application. They were fertilized three times, before water deprivation treatment, with the following solution: 20% Nitrogen (5.6% HNO_3_, 4% NH_4_^+^, 10.4% NH_2_CONH_2_), 20% P_2_O_2_, 20% K20.Fertiliser was added at 1.5 g/l to the irrigation water.

**Water treatments.** Twenty five days after sowing, irrigation on treated plants was stopped. Both well-watered (control) and water-deprived plants were weighed every day at the end of afternoon, to determine the daily transpiration (Td) of each plant. Pots were covered with a 3 mm layer of polystyrene sheet to prevent soil evaporation. Leaf area of every plant leaf was measured every two days in the morning, and stomatal conductance was calculated every day in the morning. Lost water due to transpiration was daily added to control plants, just after the pot weighting.

The soil water status was monitored using the fraction of transpirable soil water (FTSW, [101]. To estimate FTSW, a full-watering of four other pots was made and followed by one-night of drainage. Then, the initial pot weight was determined as the mean weight of the four pots. The total transpirable soil water (TTSW) is the maximum amount of available water for the plant,in each pot for this soil type. Thus, TTSW corresponds to the water held in soil between its field capacity (the water remaining in a soil after it has been thoroughly saturated and allowed to drain freely, usually for one night) and the permanent wilting point (the moisture content of the soil at which plants wilt). In our experiment, TTSW value was estimated when the stomatal conductance (gs) of the water-stressed plants in the four pots reached 10% of those of the well-irrigated plants. When this ratio was reached, the pot weight was determined and called the final pot weight. Then, TTSW was calculated as the difference between initial pot weight and the final pot weight. Mean TTSW was remarkably stable between genotypes. FTSW was then calculated by the ratio of the mass difference between daily and final pot weight to TTSW. The FTSW values of the control plants were daily brought back to 1.

For Fixed Duration Stress (FDS) implementation, plants of blocks 1 to 3 were harvested when 50% of the treated plants reached a FTSW below 0.35. This date arrived seven days after stopping irrigation, when estimated FTSW values of the treated plants ranged from 0 to 0.57 according to the genotype. For Fixed Intensity Stress (FIS) implementation, plants of blocks 4 to 6 were harvested when the treated plant reached an estimated FTSW value of 0.1±0.04. This corresponds to the Fixed Intensity Stress (FIS) subjected to plants.

### Transpiration rate

Leaf transpiration rate (E, in µg.cm^−2^.s^−1^) and stomatal conductance (gs, in cm.s^−1^) were measured from 10 a.m. with a porometer (LI-1600, Li-Cor Inc, Lincoln, NE, USA). It was measured on well-exposed and youngest expanded leaves and on abaxial face. Porometry was used to determine the dates of harvest for both fixed duration and intensity stress, allowing us to calculate TTSW value.

### Morpho-physiolocal traits

Upon each harvest (FDS and FIS), the uppermost fully expanded leaf of each plant was used to determine several morpho-physiological traits. Half the lamina of sampled leaf was used to determine Leaf Mass per Area (LMA) and Relative Water Content (RWC) .The remaining half was used to measure Leaf Osmotic Potential (OP) in order to assess osmotic adjustment.

RWC was calculated as RWC = (Fw-Dw)/(Tw-Dw), where Fw corresponds to fresh leaf weight and Tw corresponds to turgid leaf weight after 24 h rehydration at 4°C in a dark room with the petiole submerged in distilled water. Dw corresponds to dry leaf weight after subsequent oven-drying for 24 h at 80°C.

Osmotic potential (OP) at full turgor was measured on expressed sap of frozen and thawed leaves using 10 ml aliquots placed in an osmometer calibrated with manufacturer solutions (Wescor 5520, Logan, Utah, USA). Leaf osmotic potential measurements were done according to method described in detail by [97].

The leaf mass per area (LMA) was determined with discs (2 cm diameter) cut on rehydrated lamina of sampled leaves and dried (48 h, 80°C). LMA was calculated as the leaf dry weight per leaf area (m^2^.kg-1)

Carbon isotope discrimination (CID) refers to the ratio of the carbon isotopes^13^C/^12^C)in plant material, relative to the same ratio in the atmosphere. Several studies indicate that discrimination against ^13^C is proportional to plant water use efficiency [82]. In order to assess CID, the same samples of full expanded leaves used for LMA measurements were dried at 80°C. The dry samples were ground and sent to the Stable Isotope Facility at the University of Davis, CA, USA. The ground materials were analyzed for 13C isotopes using a PDZ Europa ANCA-GSL elemental analyzer interfaced to a PDZ Europa 20–20 isotope ratio mass spectrometer (Sercon Ltd., Cheshire, UK). Samples were combusted at 1000°C in a reactor packed with chromium oxide and silvered cobaltous/cobaltic oxide. Following combustion, oxides were removed in a reduction reactor (reduced copper at 650°C). The helium carrier then flowed through a water trap (magnesium perchlorate). N2 and CO2 were separated on a Carbosieve GC column (65°C, 65 mL/min) before entering the IRMS. During analysis, samples were interspersed with several replicates of at least two different laboratory standards. These laboratory standards, were selected to be compositionally similar to the samples being analyzed, and have been previously calibrated against NIST Standard Reference Materials (IAEA-N1, IAEA-N2, IAEA-N3, USGS-40, and USGS-41). A sample's preliminary isotope ratio were measured relative to reference gases analyzed with each sample. These preliminary values were finalized by correcting the values for the entire batch based on the known values of the included laboratory standards.

### Transcriptomic analysis

**Tissue harvest and RNA extraction.** In order to represent the entire plant while sparing tissue for other phenotyping procedures, every odd-numbered leaf along the whole plant was harvested for RNA extraction. Overall, between 6 and 11 leaves from each plant were pooled and then ground together. RNA extraction was performed using the Nucleo Spin RNA II extraction kit (Cat. No. 740 955.250) from Macherey-Nagel (Düren, Germany).

**The Affymetrix Sunflower Gene WT Chip.** The Affymetrix Sunflower Gene WT Chip was developed from 284 251 ESTs of seven different *Helianthus* species available at NCBI on September 27^th^ 2007. It is worth noting that even though seven *Helianthus* species were considered, *Helianthus annuus* or sunflower was the most abundantly represented species, with a total of 93 425 ESTs, i.e. 33% of the total ESTs.

The assembly of the ESTs produced 87 202 unique sequences, of which 8 378 presented ambiguous orientation, thus giving a total of 95 589 clusters that were considered for the design of the chip. Those clusters or probesets were split into ∼150 nucleotides-long Probe Selection Region, yielding a total of 397 663 PSRs. Using those PSRs, Affymetrix synthesised and spotted 2.56 million distinct, sense targeted 25-mers on the Sunflower Gene WT chip.

For our analysis we considered those probesets which contained at least one H. *annuus* EST, that is a total of 32 423 probesets comprising 897 642 probes.

**RNA labeling and Affymetrix chip hybridization.** All RNA samples were checked for their integrity on The Agilent 2100 bioanalyzer according to the specifications from Agilent Technologies (Waldbroon, Germany).

RNA concentration was measured with RiboGreen® RNA Quantification Reagent (Turner Biosystems, Sunnyvale, CA). Following Affymetrix recommendations, 100 ng of total RNA were used to synthesize fragmented and biotin-labelled single-stranded-DNAs with the GeneChip® WT cDNA Synthesis and Amplification kit and GeneChip® WT Terminal labelling kit (Affymetrix, Santa Clara, CA).

Quantity of the cRNA was determined with RiboGreen® RNA Quantification Reagent (Turner Biosystems, Sunnyvale, CA) after cleanup by the Sample Cleanup Module (Affymetrix). 15 µg of cRNA were used to obtain a single stranded cDNA, quantified with NanoDrop® Spectrophotometer ND1000 (Thermo Fisher Scientific, Waltham, MA). 5.5 µg of single stranded cDNA was fragmented and labelled followed by hybridization during 16 hours at 45°C to Affymetrix GeneChip® Sunflower genome array.

After hybridization, the arrays were washed with 2 different buffers (stringent: 6× SSPE, 0.01% Tween-20 and less-stringent: 100 mM MES, 0.1 M[Na+], 0.01% Tween-20) and stained with a complex solution including Streptavidin R-Phycoerythrin conjugate (Invitrogen/molecular probes, Carlsbad, CA) and anti Streptavidin biotinylated antibody (Vectors laboratories, Burlingame, CA). The washing and staining steps were performed in a GeneChip® Fluidics Station 450 (Affymetrix). The Affymetrix GeneChip® sunflower Genome Arrays were finally scanned with the GeneChip® Scanner 3000 7G piloted by the GeneChip® Launcher (Affymetrix).

All this steps were performed at the Affymetrix platform at INRA-URGV in Evry, France.

**Data normalization.** Raw .CEL files issued from the Affymetrix chip scanning were imported into R environment (R Foundation and Environment for Statistical Computing, Vienna, Austria)

Background noise was removed using the rma algorithm (Irizarry *et al*., 2003), available in the Affy package from Bioconductor [102]. The Intensity value of every probeset was then “block-centered” by subtracting the mean Intensity value of the probeset in a given experimental block to the Intensity of that probeset in every chip. The presence of negative Intensity values was avoided by adding up the global mean Intensity value for the probeset in all 96 individuals. Subsequently, quantile normalization was carried out using the normalize.quantiles function available in the preprocessCore package from Bioconductor.

All raw and normalized data are available through the CATdb database (AFFY_Sunyfuel_drought_Sunflower and AFFY_TOUR_2010_21, [103] and from the Gene Expression Omnibus (GEO) repository at the National Center for Biotechnology Information (NCBI, [104]: accession number GSE25719.and GSE 36 304

**Data Analysis.** Data analysis was performed in R environment and scripts are available upon request. ANOVAs were carried out in order to determine differentially expressed genes under block, treatment, genotype and genotype*treatment effects. Mean common residual variance was applied to probesets sharing homoscedastic values in our model, as tested by Bartlett's test. FWER-type error due to multiple tests was controlled to 5% using Bonferroni's procedure. *No gene presented block effect and thus, this effect has not been discussed in the manuscript.*

SPLS covariance analysis was achieved using the *mixOmics* package available in Bioconductor [79], [102]. This projection-based method is particularly adapted when the number of variables exceeds the number of individuals. SPLS combines linear combinations between two datasets and LASSO-type penalization in order to discriminate relevant combinations, with increases biological interpretability. We used SPLS in regression mode, which modelizes causal relationships, thus predicting physiological responses out of transcriptomic data.

*GO term enrichment tests were performed using Singular Enrichment Analysis (SEA) on the AgriGO website* [105] *by comparing the annotations of Arabidopsis homologs to a subset of sunflower transcripts, with the annotations of Arabidopsis homologs of all sunflower transcripts present on the chip. We performed hypergeometric tests with FDR under dependency for multi-test adjustment.*

For enrichment tests of genes related to a given physiological trait in the network, we performed a hypergeometric test *using the function hygepdf.m in the Matlab Statistical toolbox (v7.4).*

**qRT-PCR validation.** *Gene expression validation was conducted via the* BioMark™ HD System using 96.96 digital array chips from Fluidigm Corporation [106]

Genes showing sharp fold changes (FC) in the Affymetrix chip, presenting either up- or down-regulation under water deprivation for every genotype in the design, were chosen and tested for qRT-PCR validation. A total of 10 genes were chosen and validated. For this purpose, sunflower reference genes were chosen among the genes presenting no modulation under water deprivation according to the results obtained from the Affymetrix hybridizations. That is, genes showing FC = 1 in every genotype and smallest standard deviation among individuals. A total of 30 reference genes were initially picked and eight of them, i.e. those presenting the smallest variability among individuals according to the qRT-PCR results and sharing similar Ct values with the tested genes, were finally retained for the analysis of the tested genes. The mean expression value of those eight genes was used in every individual in order to normalize the expression values of the 10 tested genes. Ninety five individuals out of the 96 in the design were tested in the same Biomark array. Boxplots showing the qRT PCR results for the reference genes as well as the correlation between the Affymetrix results and the qRT-PCR results for the tested genes are shown in Figure S5.

**Experimental Design in the field.** Plants were grown at INRA in Auzeville-Tolosane (Haute-Garonne, France). They were sown on the 7^th^ of May 2009 and the tissue used for the chip hybridizations was harvested on 30^th^ July 2009, that is 85 days after sowing, and approximately 10 days after flowering.

The assay was arranged in plots of 4 rows separated by 50 cm, each having circa 24 plants separated by 25 cm, with one genotype per plot. It was divided in two identically seized parts, one irrigated and the other one not. Four plants from four plots of MELODY (two per treatment) were selected. Plots were included in a larger trial and spread randomly across the field capturing most of its heterogeneity. Starting from the head, leaf −3 was harvested for subsequent grinding and RNA extraction.

A basic water balance model was used to decide when to harvest plants. Tissues were harvested when the ratio between the actual evapotranspiration and the maximal evapotranspiration, as calculated by BILH model [107], was 0.63 and 0.22 in the irrigated assay and the non-irrigated assay respectively. This corresponded to an optimal difference between treatment i.e. to a mild and severe stress in agronomic conditions.

## Supporting Information

Figure S1 **Distribution of the number of probes per probeset in the** ***Helianthus annuus*** **probesets from the sunflower Affymetrix microarray.**
(TIF)

Figure S2 **Results of the sparse Partial Least Squares (SPLS) analysis on plants under FDS (A) and FIS (B).** Plots show respectively the repartition of the morphophysiological variables (left) and individuals (right) along the first three components of the SPLS. Morphophysiological variables are carbon isotope discrimination (CID), collar diameter (CoD), Transpiration rate (E), Integrated Transpired Water (ITW), Leaf Mass per Area (LMA), Osmotic Potential (OP), Plant Height (PHe), Relative Water Content (RWC) and Total plant Leaf Area (TLA). Triangles correspond to treated plants whereas circles correspond to their untreated counterparts. Genotypes are color-coded as follows: Inedi (black), Tekny (gray), Melody (red), SF109 (turquoise), SF326 (yellow), SF193 (magenta), SF028 (green) and SF107 (blue).
(TIF)

Figure S3 **Gene-Phenotype network produced by SPLS in the FDS scenario, based on responses of eight sunflower genotypes to two drought stress scenarios implemented in controlled environment.** Genes presenting absolute correlation scores higher than 0.65 with at least one morpho-physiological variable are represented. Each circle represents one gene. Red and blue edges indicate, respectively, whether the gene-phenotype correlation is respectively positive or negative. Each gene circle is colored according to the ANOVA effect associated to the gene. Phenotypic responses are in gray squares, OP: Osmotic Potential, LMA: Leaf Mass Area, E: Transpiration Rate, RWC: Relative Water Content, ITW: Integrated Transpired Water, PHe: Plant Height.
(TIF)

Figure S4 **Gene-Phenotype network produced by SPLS in the FIS scenario, based on responses of eight sunflower genotypes to two drought stress scenarios implemented in controlled environment.** Genes presenting absolute correlation scores higher than 0.65 with at least one morpho-physiological variable are represented. Each circle represents one gene. Red and blue edges indicate, respectively, whether the gene-phenotype correlation is respectively positive or negative. Each gene circle is colored according to the ANOVA effect associated to the gene. Phenotypic responses are in gray squares, OP: Osmotic Potential, LMA: Leaf Mass Area, E: Transpiration Rate, RWC: Relative Water Content, ITW: Integrated Transpired Water, PHe: Plant Height.
(TIF)

Figure S5 **Scatter plots and correlations between the Affymetrix microarray intensities and the q-RTPCR results.** Genotypes are color-coded as follows: Inedi (black), Tekny (gray), Melody (red), SF109 (turquoise), SF326 (yellow), SF193 (magenta), SF028 (green) and SF107 (blue).
(TIF)

Table S1 **Phenotypic data of each sunflower plant in FDS and FIS in greenhouse experiment.**
(XLS)

Table S2 **Sunflower Affymetrix probeset annotations and statistical test results for the ANOVA analysis in FDS and FIS.**
(CSV)

Table S3 **Numbers of transcripts associated to the different phenotypic varibles in the Gene-Phenotype network obtained through the sPLS analysis.**
(XLS)

Table S4 **Sunflower Affymetrix probesets regulated by drought stress in field condition and their annotations, links to phenotypic variables in the Gene-Phenotype network, their GO term assiciated and the GO term enrichment test results.**
(XLS)
