# Supplementary figures and images for: A Gene-Phenotype Network Based on Genetic Variability for Drought Responses Reveals Key Physiological Processes in Controlled and Natural Environments
Source: PLoS One. 2012 Oct 8;7(10):e45249. doi: 10.1371/journal.pone.0045249 (PMC3466295; doi:10.1371/journal.pone.0045249)

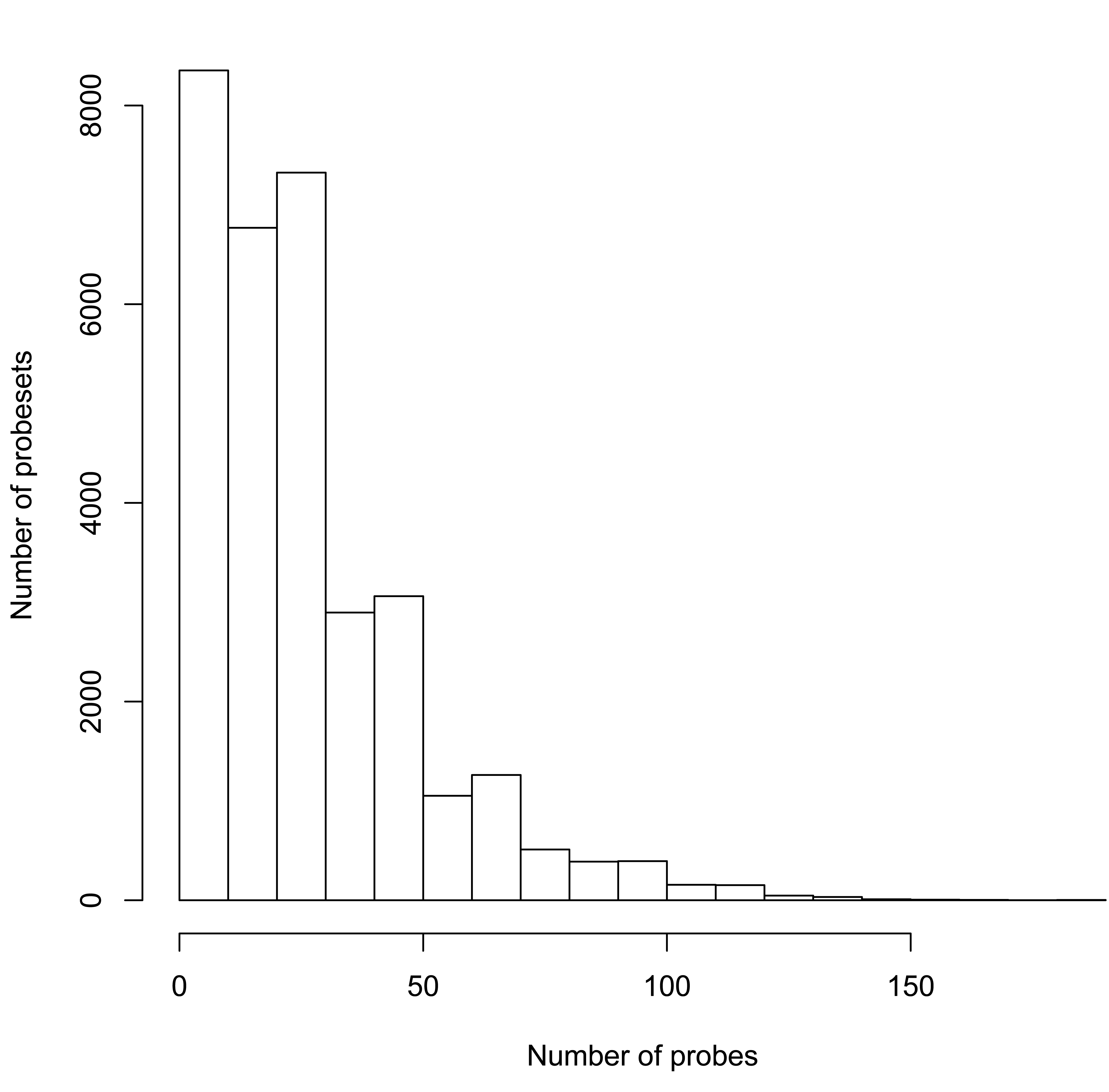

Supplement: Figure S1 — Distribution of the number of probes per probeset in the Helianthus annuus probesets from the sunflower Affymetrix microarray. (TIF) [file pone.0045249.s001.tif]

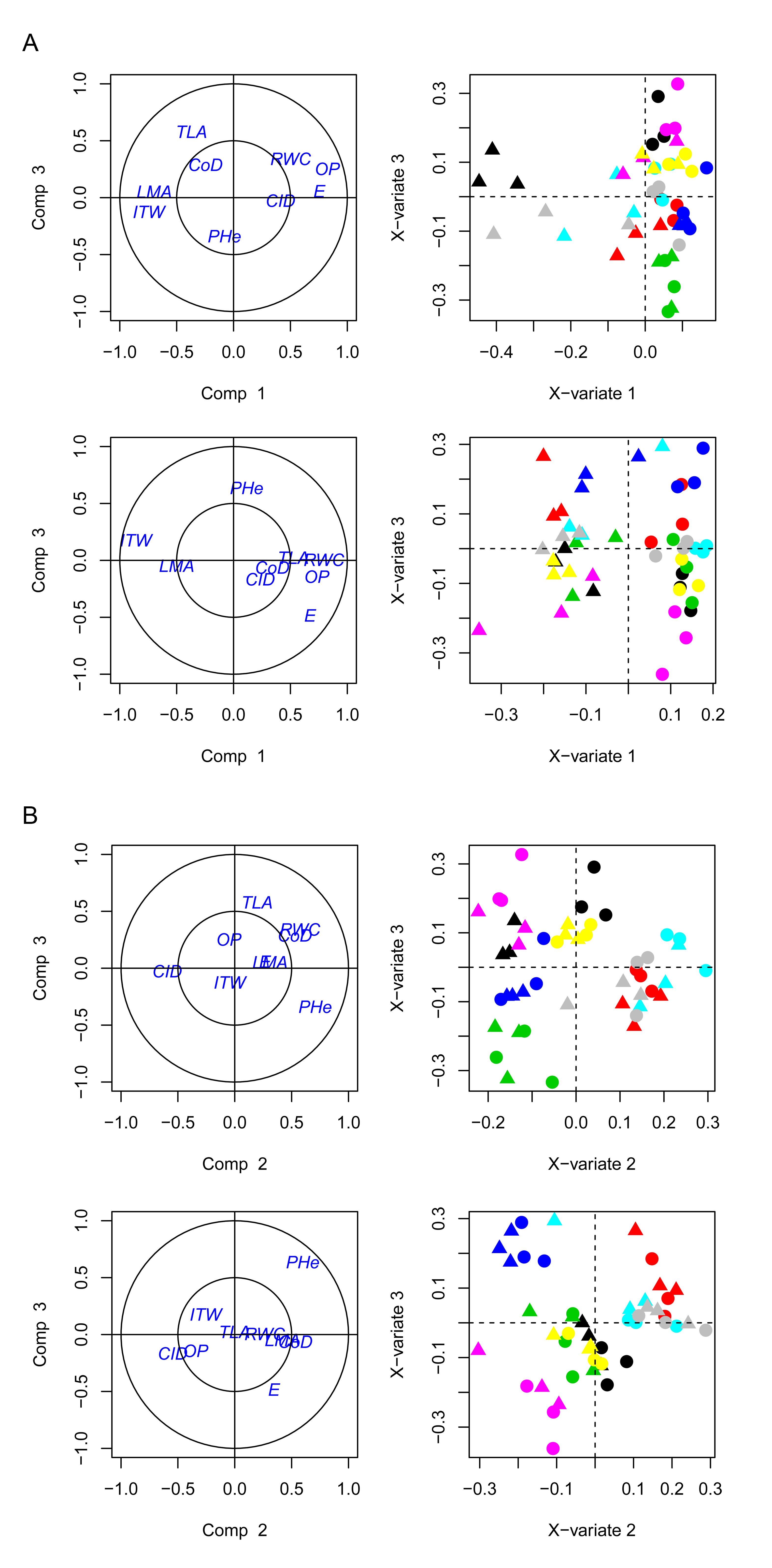

Supplement: Figure S2 — Results of the sparse Partial Least Squares (SPLS) analysis on plants under FDS (A) and FIS (B). Plots show respectively the repartition of the morphophysiological variables (left) and individuals (right) along the first three components of the SPLS. Morphophysiological variables are carbon isotope discrimination (CID), collar diameter (CoD), Transpiration rate (E), Integrated Transpired Water (ITW), Leaf Mass per Area (LMA), Osmotic Potential (OP), Plant Height (PHe), Relative Water Content (RWC) and Total plant Leaf Area (TLA). Triangles correspond to treated plants whereas circles correspond to their untreated counterparts. Genotypes are color-coded as follows: Inedi (black), Tekny (gray), Melody (red), SF109 (turquoise), SF326 (yellow), SF193 (magenta), SF028 (green) and SF107 (blue). (TIF) [file pone.0045249.s002.tif]

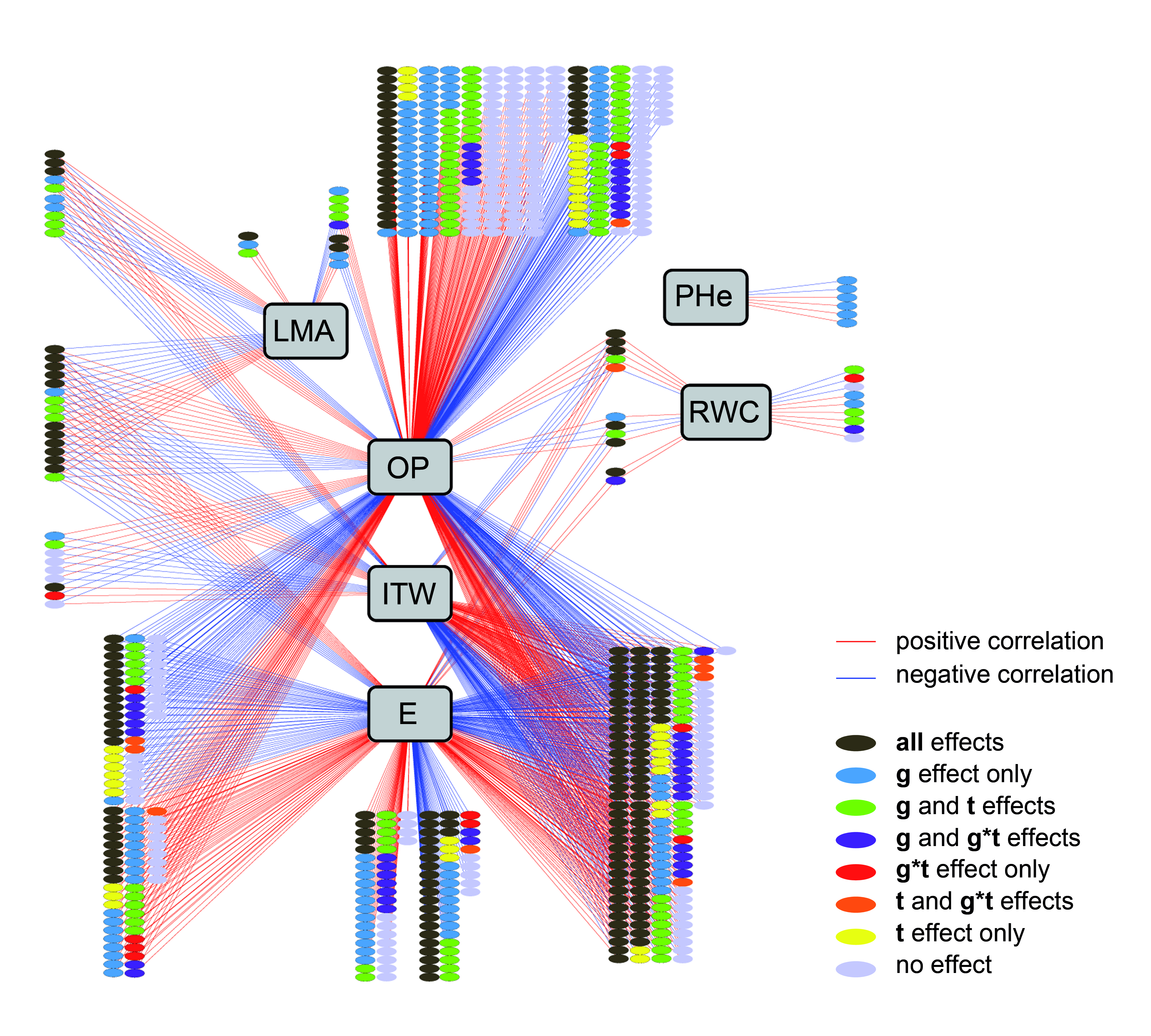

Supplement: Figure S3 — Gene-Phenotype network produced by SPLS in the FDS scenario, based on responses of eight sunflower genotypes to two drought stress scenarios implemented in controlled environment. Genes presenting absolute correlation scores higher than 0.65 with at least one morpho-physiological variable are represented. Each circle represents one gene. Red and blue edges indicate, respectively, whether the gene-phenotype correlation is respectively positive or negative. Each gene circle is colored according to the ANOVA effect associated to the gene. Phenotypic responses are in gray squares, OP: Osmotic Potential, LMA: Leaf Mass Area, E: Transpiration Rate, RWC: Relative Water Content, ITW: Integrated Transpired Water, PHe: Plant Height. (TIF) [file pone.0045249.s003.tif]

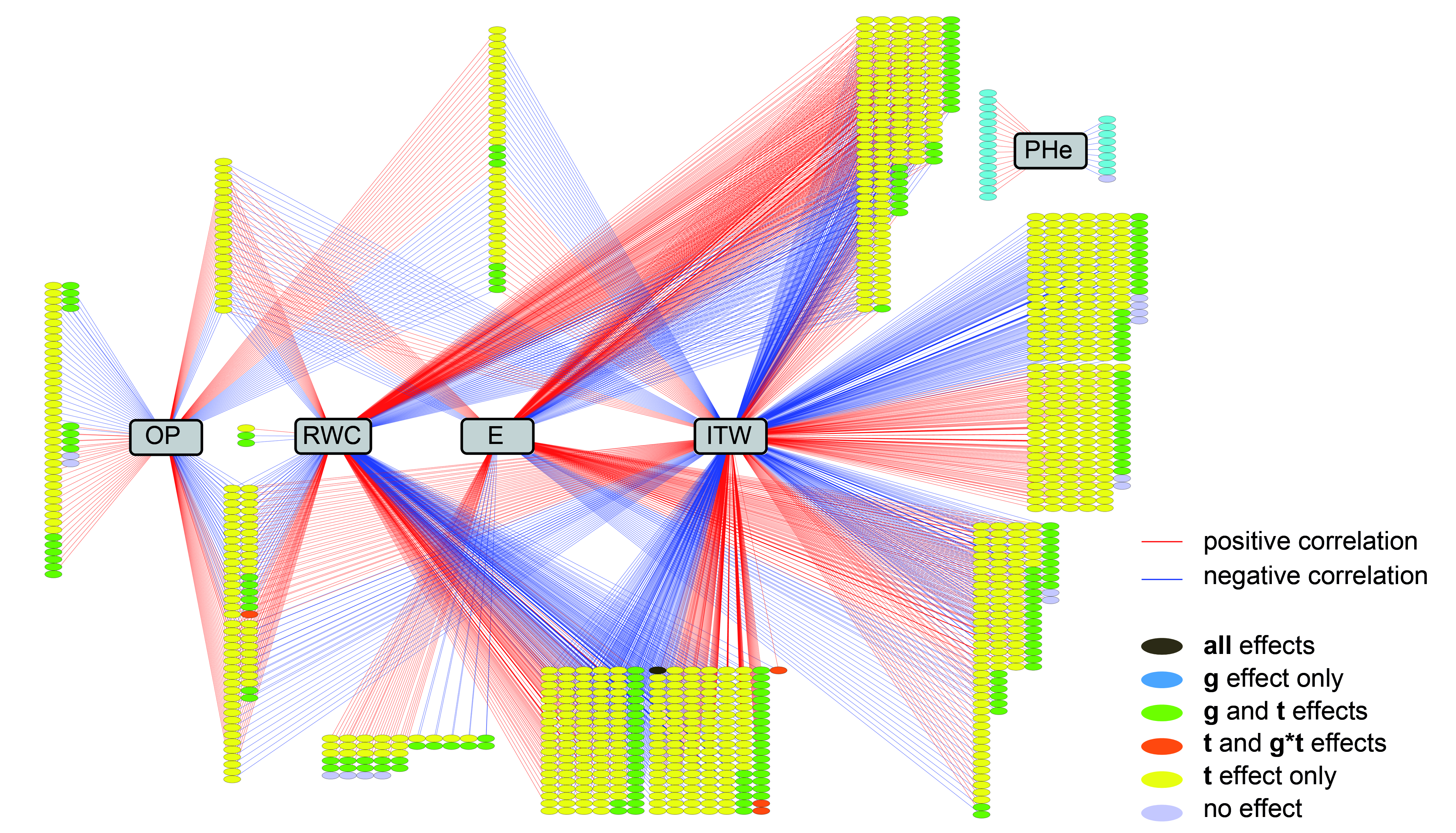

Supplement: Figure S4 — Gene-Phenotype network produced by SPLS in the FIS scenario, based on responses of eight sunflower genotypes to two drought stress scenarios implemented in controlled environment. Genes presenting absolute correlation scores higher than 0.65 with at least one morpho-physiological variable are represented. Each circle represents one gene. Red and blue edges indicate, respectively, whether the gene-phenotype correlation is respectively positive or negative. Each gene circle is colored according to the ANOVA effect associated to the gene. Phenotypic responses are in gray squares, OP: Osmotic Potential, LMA: Leaf Mass Area, E: Transpiration Rate, RWC: Relative Water Content, ITW: Integrated Transpired Water, PHe: Plant Height. (TIF) [file pone.0045249.s004.tif]

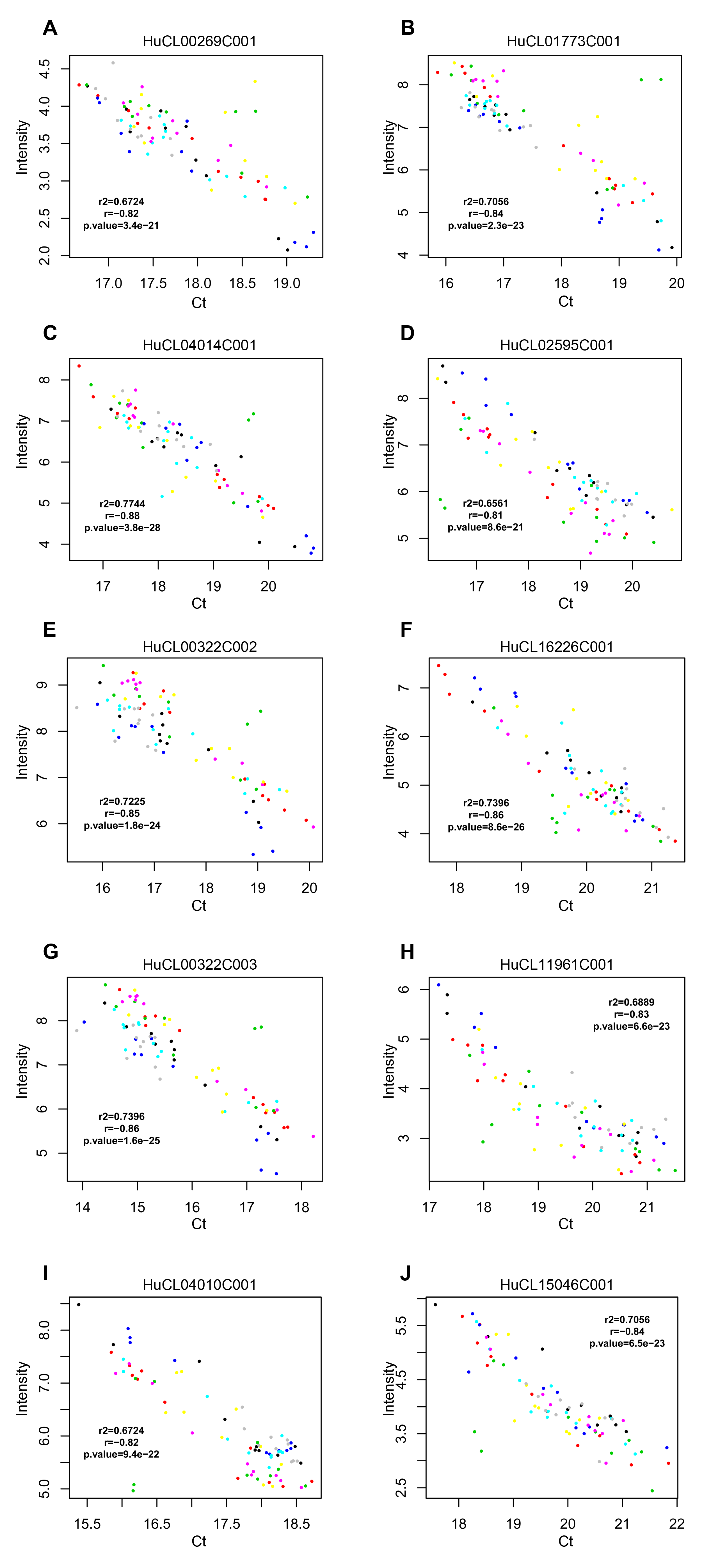

Supplement: Figure S5 — Scatter plots and correlations between the Affymetrix microarray intensities and the q-RTPCR results. Genotypes are color-coded as follows: Inedi (black), Tekny (gray), Melody (red), SF109 (turquoise), SF326 (yellow), SF193 (magenta), SF028 (green) and SF107 (blue). (TIF) [file pone.0045249.s005.tif]
